# CHO stable pool fed‐batch process development of SARS‐CoV‐2 spike protein production: Impact of aeration conditions and feeding strategies

**DOI:** 10.1002/btpr.3507

**Published:** 2024-09-27

**Authors:** Sebastian‐Juan Reyes, Phuong Lan Pham, Yves Durocher, Olivier Henry

**Affiliations:** ^1^ Department of Chemical Engineering Polytechnique Montreal Quebec Canada; ^2^ Human Health Therapeutics Research Centre National Research Council Canada Montréal, Quebec Canada

**Keywords:** bio‐capacitance, CHO stable pool, fed‐batch process development, feeding strategy, oxygen uptake rate, SARS‐CoV‐2 vaccine antigen

## Abstract

Technology scale‐up and transfer are a fundamental and critical part of process development in biomanufacturing. Important bioreactor hydrodynamic characteristics such as working volume, overhead gas flow rate, volumetric power input (P/V), impeller type, agitation regimen, sparging aeration strategy, sparger type, and k_L_a must be selected based on key performance indicators (KPI) to ensure a smooth and seamless process scale‐up and transfer. Finding suitable operational setpoints and developing an efficient feeding regimen to ensure process efficacy and consistency are instrumental. In this investigation, process development of a cumate inducible Chinese hamster ovary (CHO) stable pool expressing trimeric SARS‐CoV‐2 spike protein in 1.8 L benchtop stirred‐tank bioreactors is detailed. Various dissolved oxygen levels and aeration air caps were studied to determine their impact on cell growth and metabolism, culture longevity, and endpoint product titers. Once hydrodynamic conditions were tuned to an optimal zone, various feeding strategies were explored to increase culture performance. Dynamic feedings such as feeding based on current culture volume, viable cell density (VCD), oxygen uptake rate (OUR), and bio‐capacitance signals were tested and compared to standard bolus addition. Increases in integral of viable cell concentration (IVCC) (1.25‐fold) and protein yield (2.52‐fold), as well as greater culture longevity (extension of 5 days) were observed in dynamic feeding strategies when compared to periodic bolus feeding. Our study emphasizes the benefits of designing feeding strategies around metabolically relevant signals such as OUR and bio‐capacitance signals.

## 1 INTRODUCTION

Currently, mammalian cell lines like Chinese Hamster Ovary (CHO) cells are the industry standard for monoclonal antibody and recombinant therapeutic protein (RTP) production because these organisms can be adapted to produce human‐like proteins through various transient or stable gene expression strategies. Developing stable cell lines can often be a time‐consuming process that requires numerous screening procedures to differentially select the best behaving cell clones.^1^ This can clearly be an issue if the desired biotherapeutic product is urgently needed as a response to a rapidly changing public health crisis such as the one caused by the SARS‐CoV‐2 Covid 19 pandemic.^2^ Therefore, mammalian cell pools have been considered as an interesting option to accelerating biotherapeutic production processes that do not require several months of screening and selection experiments to provide enough material for toxicology studies and Phase 1 clinical trials. Such cell pools, although inherently not as homogeneous in terms of cell population, can be employed to produce target proteins on a large scale.^3^ Importantly, clones selected for production during the high‐throughput screening phase are not necessarily guaranteed to work at larger scales due to varying culture environment and conditions, thus there is a case to be made to diminish excessive resource utilization at micro liter scale and instead shift the focus towards representative scalable models. Here, cell pools can be utilized to fast forward to benchtop bioreactor scale in order to find adequate critical process parameters (CPPs) that are more representative of large‐scale stirred tank bioreactor production. These pools can be affected by cell age, such that target protein expression productivity may significantly diminish with increased cell passage number.^4^ Thus, for stable pools to overcome their disadvantages and be a clear alternative to clonally derived cell lines, the production phase needs to start as soon as possible and the process itself has to be highly optimized.

Furthermore, the metabolism of mammalian cells is dependent on prevailing process conditions. Thus, unoptimized process parameters (e.g., suboptimized feeding strategies) can generally cause overflow metabolism in which inhibitory by‐products are accumulated within the bioreactor causing the cells to lose prematurely viability and productivity.^5^ Fed‐batch is the dominant process used in biomanufacturing mostly due to its simplicity and efficacy. Feed bolus addition is largely developed and employed at large‐scale. However, possible nutrient limitation or accumulation of primary inhibitor metabolites can induce early culture crash in large bioreactors. Feed on‐demand can be an alternative option to avoid premature culture failure. For this to be possible, continuous streams of data regarding media composition or cellular metabolic activity can be key when constructing dynamic feeding strategies, which are automatically triggered when important nutrients are becoming limiting. Given this knowledge, instead of feeding cultures based on a predefined schedule, feeding strategies should be triggered or set based on biologically relevant measurements that vary over time and from culture to culture.^6^ Due to the metabolic complexity of mammalian cells, recent trends have evolved towards developing and optimizing soft sensors that can abstract information from various sensor sources rather than one single measurement.^6^ For example, feeding based on the integral of the bio‐capacitance signal has been developed as a way to adjust feed rates with varying biomass.^7^ Since bio‐capacitance signals have a strong correlation with viable cell volume, such feeding has the added value of taking into account changes in cell size typically observed during cultures.^8^, ^9^, ^10^ With this strategy, automation of feeding protocol regardless of initial seeding density or bioreactor scale can be achieved. Bio‐capacitance signals have also been used in conjunction with glucose measurements to estimate specific glucose consumption rate in real time that can then be used to forecast glucose consumption and supplement accordingly to diminish substrate variations.^11^ It is worth noting that alterations in cell membrane properties are also believed to potentially impact this parameter.^12^ Nutrient availability has also been found to be monitored through permittivity measurements because, at every feeding event, permittivity changes were detected to increase while declines in permittivity signals were correlated to states of nutrient depletion.^12^ Additionally, during the exponential phase (which is when cell radius remains more or less constant), good correlations with oxygen uptake rate (OUR) were obtained. This may suggest that metabolic activity may also be indirectly reflected by dielectric spectroscopy.^12^

Other approaches have centered around measurement of the OUR due to the strong linear correlation between oxygen consumption and viable cell density. OUR has thus been used to dictate glucose additions based on estimated viable cell densities^13^ allowing to control glucose concentrations near a desired setpoint without large deviations. It has been postulated that the linear correlation between the cumulative oxygen consumption rate and the cumulative glucose consumed during the production phase can be used within a control strategy to maintain glucose level in the media at a given setpoint.^14^, ^15^ Such strategies were observed to diminish glucose concentration variations (underfeed or overfeed) which are inevitably observed with bolus additions. OUR signals can also be used to detect nutrient limitations given that spent media analysis revealed that decreases in respiratory rates were correlated with exhaustion of key amino acids. Thus, OUR can be used to supplement not only glucose but other important nutrients so as to optimize overall culture performance.^16^ It has been observed that, during the protein production phase, a metabolic shift occurs such that the tricarboxylic acid cycle (TCA) is upregulated and thus cells are subjected to increased oxidative stress, suggesting that a highly oxidative state of metabolism corresponded to peak antibody production while a highly glycolytic state corresponded to peak growth cycles.^17^, ^18^ OUR can continue to increase even after peak viable cell densities are obtained indicating that a relationship exists between volumetric oxygen demand and volumetric protein production.^19^ Specific oxygen consumption rates and specific protein production rates seem to have a direct relationship, suggesting a close physiological connection among cellular respiration and product formation rates.^16^ Other studies also showed a linear relationship between the energy production rate and OUR.^20^, ^21^ Moreover, it was concluded that energy production rate is in positive relation with recombinant protein production rate.^22^ Thus, the specific OUR can be used to represent the activity of the TCA cycle and the energy metabolic state of the cells. High values of specific OUR in the recombinant protein production phase can suggest high specific ATP production rate through TCA cycle (if the phosphate/oxygen ratio is almost constant). This, in turn, can lead to the increase in the specific recombinant protein production rate.^20^ Taken together, estimating OUR in real time can be beneficial for real time monitoring of cell culture performance.

Reliable estimation of OUR can be challenging and is usually performed through one of the following three standard methods: the dynamic method, the global mass balance (GMB) method, and the stationary liquid mass balance (LMB).^23^ The dynamic method relies on the cyclical measurement of the DO extinction profile when air supply is stopped. The DO concentration decreases due to cellular oxygen need and thus OUR is directly proportional to the slope of the decay curve. The GMB approach relies on estimating the oxygen concentrations at the inlet and outlet of the bioreactor while the DO is kept constant such that the oxygen transfer rate (OTR) is equal to OUR. Mass spectrometers, paramagnetic sensors or acoustic analyzers can be used.^23^ The liquid mass balance (LMB) method depends on the estimation of $k_{L}a$ in real time which can be difficult given that changes in gas flow rates or stirring (common parameters for DO control) can change its value.^23^ Of note, it has been found that even though OUR is strongly linked with viable cell density, viable cell volume can lead to more precise correlations with OUR^24^, ^25^ as larger cells can have increased oxygen requirements when compared to smaller cells.^26^ Importantly, as the cell diameter increases, the total biovolume of the culture increases while the cell count stays constant. This biovolume increase is reflected in both OUR demand and bio‐capacitance measurements, but not on cell counts.

The framework presented in this article centers around technology transfer from a 0.75 L (1 L total volume) Multifors 2 bioreactor (Infors) to a 1 L (1.8 L total volume) DASGIP multisystem bioreactor (Eppendorf) of an inducible CHO stable cell pool and its fed‐batch production process that can manufacture the SARS‐CoV‐2 spike protein as a potential vaccine antigen. Inducible CHO stable pools have recently been shown to robustly express SARS‐CoV‐2 spike protein thanks to the cumate gene switch system derived from the cymene operon of Pseudomonas putida.^3^, ^27^ Briefly, rCymR is fused with an activation domain (VP16) to form the reverse cumate activator (rcTA) that induces transcription when cumate is present, as opposed to when it is absent.^28^ Thus, cumate changes the conformation of the chimeric molecule. When no cumate is present, rcTA is not able to bind to the operator sites, while in the presence of cumate, rcTA is able to properly bind to the operator binding sites and thus initiate activation. In general, bioreactor processes are characterized by scale‐dependent and scale‐independent parameters. Scale‐independent parameters like temperature, pH, and substrate concentrations, are, in principle, parameters that can be matched across process scales.^29^ It is known that the key to realizing process transfer is to maintain scale‐independent process characteristics constant across systems. Conversely, scale‐dependent parameters can vary considerably with reactor size and configuration. Therefore, process technology transfer is to find out which scale‐dependent variable should be kept constant and more importantly, to determine a good design space for the other scale‐dependent variables which is not adverse to the culture.^29^ In industrial applications, the most common scale‐up and process transfer strategies are constant P/V, constant 𝑘_𝐿_𝑎, and constant impeller tip speed.^30^ Constant P/V criteria have been used successfully to transfer a base‐free ambr15 process to a 200 L pilot scale.^31^ Additionally, this strategy has also shown to produce similar growth, productivity, and glycosylation profiles across various scales (ambr15, 3, 50, and 500 L).^32^, ^33^ It must be noted that constant P/V can translate to a large difference in the $k_{L}a$ values between two scales and as such, alterations in the gassing profiles may be required to maintain the DO setpoint.^34^

Within the context of our study, we aimed to delve the effect of aeration strategies and feeding regimens on overall process performance. The key process transfer strategy used was keeping an equal P/V range across both systems (Multifors 2 and DASGIP parallel bench‐scale bioreactors) and ensuring tip speed was kept below a threshold of 1 m/s as suggested in the literature for working volumes below 1 L.^29^ Overhead flow rates were chosen such that a constant flow with respect to initial volume is subjected to the bioreactors (0.033 vvm) to avoid carbon dioxide accumulation and to replicate the overhead flow of the 0.75 L Multifors 2 system.^35^, ^36^, ^37^ Another relevant parameter is the dissolved oxygen (DO) setpoint. This value must provide the adequate oxygen availability in the medium for cells to consume during their metabolic activity and thus is expected to decrease as biomass grows.^38^ This relates to the importance of constantly supplementing oxygen such that it is controlled around a setpoint. If this value was to be critically low (5% DO), alterations in the ratio of glucose consumption and lactate production have been observed.^39^ Conversely, high DO setpoints (200% DO) can lead to the accumulation of reactive oxygen species such that a negative impact on cell growth is detected^40^ which implies that finding the adequate range of DO operation is key. Even though fed‐batch strategies are the industry standard,^6^ bolus feeding‐induced oscillations on CHO cell cycles have been noted which were caused by variations in nutrient concentrations. This suggests that alternatives to standard bolus practices could be explored.^41^, ^42^

Taken together, optimizing nutrient supplementation and gas transfer conditions within the bioreactor are two critical processes that must be carried out to obtain a robust manufacturing platform. Our research shows that synergistic effects of feeding and hydrodynamics must be considered and thus, future optimization must be done simultaneously given the strong impact that both processes have on culture outcomes.

## 2 MATERIALS AND METHODS

### 2.1 Bioreactor cell culture conditions

A cumate‐inducible proprietary CHO‐GS stable cell pool expressing SARS‐CoV‐2 trimeric spike protein (SmT1) was grown in BalanCD CHO Growth A medium (Fujifilm/Irvine Scientific) supplemented with 50 μM MSX (L‐Methionine sulfoximine, Sigma‐Aldrich) in 1.8 L (initial working volume of 650 mL and a maximum working volume of 1100 mL) benchtop bioreactors (DASGIP parallel bioreactor system, Eppendorf). Corning shake flasks without baffles were used to generate seed trains. The flasks were shaken at 180 rpm (25 mm orbital diameter) in a ThermoFisher HERAcell 240i incubator with 5% ${\mathrm{CO}}_{2}$ and 75% relative humidity. The bioreactors were seeded at $0.4\times 10^{6}$ cells/mL and cultivated for 17 days. Temperature downshift (37–32°C) was realized 3 days after seeding unless stated otherwise. A pH shift was also performed on all bioreactors 2 days after seeding (from 7.05 ± 0.05 to 6.95 ± 0.05). Induction was realized with 4‐isopropylbenzenecarboxylate (Cumate, ArkPham) 3 days post‐seeding. Cultures were fed with BalanCD CHO Feed 4 (Fujifilm/Irvine Scientific) and supplemented with glucose to maintain the concentration above 17 mM (3 g/L) on the next sampling and feeding event. 6 mL samples were taken from the bioreactors on days −3, −2, −1, 0, 3, 5, 7, 10, 12, and 14 dpi (day post‐induction) for off‐line analysis, while feeding was realized from 0 dpi (3 days post‐seeding) onward unless stated otherwise. Metabolic measurements (residual glucose, lactate, ammonia) were performed using the Cedex Bio (Roche, Switzerland). The manual cell counts using a hemocytometer were observed to have an average relative standard deviation error of 8%. Titer estimation using the SDS‐PAGE TGX (BioRad) gel method had an average relative standard deviation error of 12%. Amino acid measurements were conducted following the AccQ‐Tag Ultra Derivatization Kit protocol (Waters Corporation, USA) that employs an AccQ‐Tag Ultra C18 column with the ACQUITY H‐Class UPLC system (Waters Corporation, USA) and UV detection. Offline osmolarity measurements of supernatant samples were realized with osmoTECH from Advanced instruments. Culture permittivity and conductivity were measured online using the Aber Futura biomass capacitance probe which was calibrated to 0 using fresh media prior to seeding to track biomass across the culture run. An open pipe sparger (4 mm outer diameter, 2 mm inner diameter) was used in all the bioreactor conditions. The overhead flow was set at 0.033 VVM (Gas Volumetric Flowrate in L/H divided by Initial Liquid Volume in L). Agitation was set at 250 rpm utilizing a single 45° pitch‐blade impeller to maintain a volumetric power input (P/V) within the range of 20–30 $W/m^{3}$.

The static gassing out method was employed to estimate oxygen $k_{L}a$ values. First oxygen concentration was reduced to zero by nitrogen degassing. Then, gassing was reintroduced under process specific conditions. An optical Hamilton dissolved oxygen (DO) sensor recorded the saturation process, enabling the determination of $k_{L}a$ values. OTR was estimated through a BluSenses off‐gas analyzer GmbH utilizing the global mass balance (GMB) approach which relies on estimating the oxygen concentrations at the inlet and outlet of the bioreactor while the DO is kept constant.

$$\mathrm{OTR}=\frac{G_{m}\times \left({\mathrm{yO}}_{2,\text{in}}-{\mathrm{yO}}_{2,\text{out}}\right)}{V_{L}}$$

OTR (mmol/(L*H)) represents the oxygen transfer rate, $G_{m}$ is the total gas flow rate (L/H), ${\mathrm{yO}}_{2,\text{in}}$ is the oxygen concentration (mol/L) at the inlet, ${\mathrm{yO}}_{2,\text{out}}$ is the oxygen concentration (mol/L) at the outlet, and $V_{L}$ is the total bioreactor volume.^23^ Variation in volume caused by feeding and sampling is taken into account during the estimation of OTR.

Online raw signals (OUR, capacitance, gas flows) were treated using Savitzky–Golay filtering in R to reduce noise in the data. Data pre‐processing, analysis, and visualization were carried out in R.^43^

### 2.2 Study schematic

**FIGURE 1:**
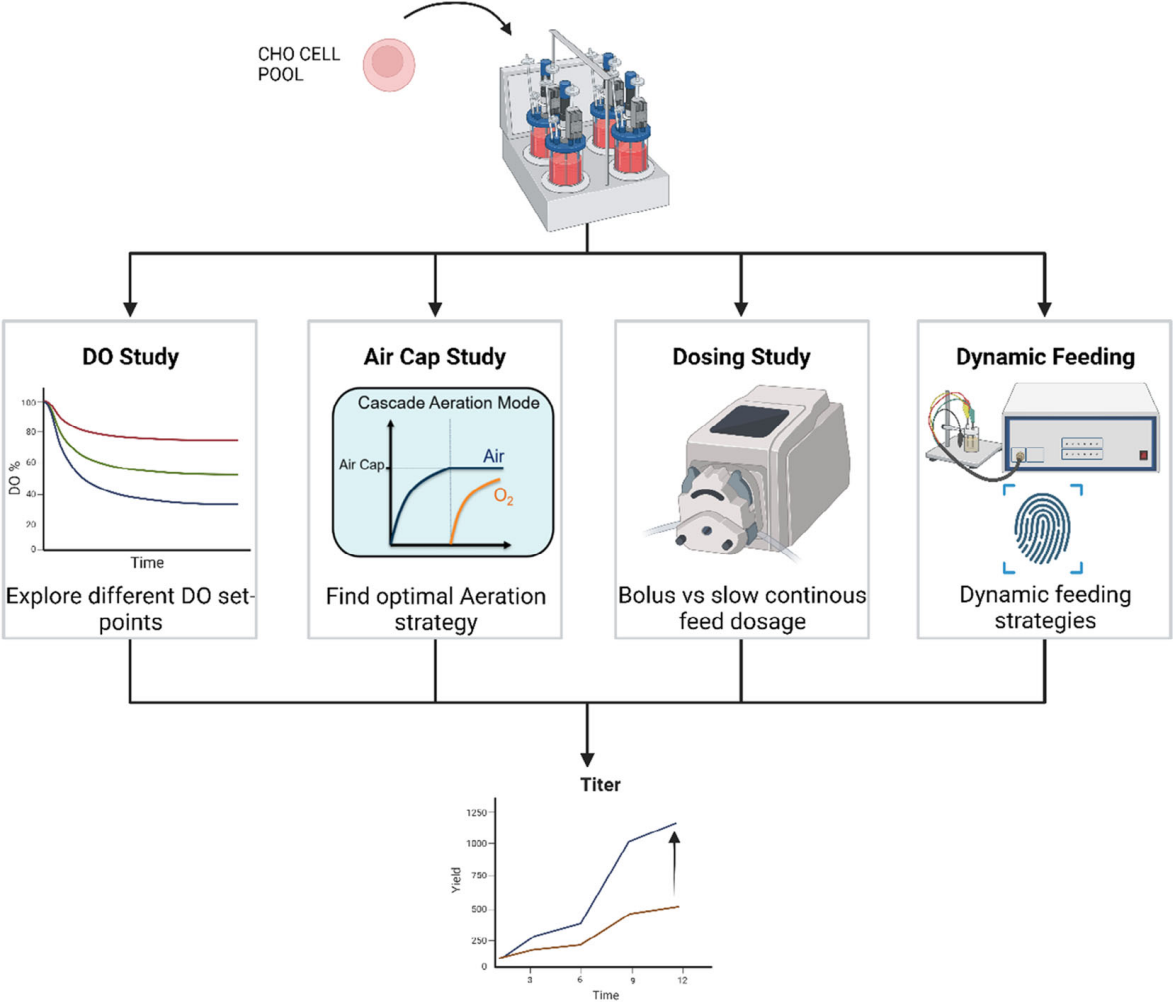
Experimental workflow.

Figure 1 summarizes the studies performed in the process transfer stage of SARS‐CoV‐2 spike protein production. As mentioned above, the initial process has been established in the 0.75 L Multifors 2 (Infors) bioreactors.^3^ This process was transferred to the 1 L DASGIP (Eppendorf) presented in this article. Two studies on aeration strategies have been conducted investigating the impact of DO and air cap optimization. Further improvement of production performance has been enabled through two subsequent experiments delving the impact of feeding strategy.

DO study allowed to find optimal dissolved oxygen tension setpoint. Air cap study allowed to determine best performing aeration strategy while also further confirming DO setpoints. Dosing study determined the impact on culture outcomes when comparing bolus addition of feed versus slow pump feeding. Dynamic feeding study demonstrated feasibility of setting feed flow rates to track sensor signals.

The details of studied conditions are displayed in Table 1.

**TABLE 1:** Experimental conditions.

Experiment number	Temperature shift day	Air cap (L/H)	Dissolved oxygen, %	Feeding mode	Dosage
1	0 dpi	1.5	90	FFIV	Bolus
2	0 dpi	1.5	60	FFIV	Bolus
3	0 dpi	1.5	40	FFIV	Bolus
4	0 dpi	2.25	40	FFIV	Bolus
5	0 dpi	3	40	FFIV	Bolus
6	0 dpi	1	60	FFCV	Slow pump
7	0 dpi	3	60	FFCV	Slow pump
8	0 dpi	5	60	FFCV	Slow pump
9	3 dpi	1.5	60	FFIV	Slow pump
10	3 dpi	1.5	60	FFCV	Slow pump
11	0 dpi	1.5	60	FFCV	Bolus
12	0 dpi	1.5	60	FFIV	Bolus
13	2 dpi	1.5	60	CAP	Slow pump
14	2 dpi	1.5	60	CFPC	Slow pump
15	2 dpi	1.5	60	OTRF	Slow pump
16	0 dpi	1.5	60	FFIV	Bolus (Control process)

Abbreviations: CAP, capacitance based feeding; CFPC, constant feed per cell‐based feeding; FFCV, fixed feeding based on current volume; FFIV, fixed feeding based on initial volume; OTRF, OTR based feeding.

### 2.3 Study of DO level impact

In this study, the air sparged flow rate was increased according to the cell growth until a limit (air cap of 1.5 L/H) followed by oxygen sparging as needed. Air caps were kept constants for all conditions while DO setpoints were varied (Table 1). Three concentrations of DO (40%, 60%, 90%) have been chosen.

### 2.4 Air caps and increased feed study

To evaluate the impact of air caps at a given DO level, a range between 1 and 5 L/H (0.0015–0.0077 vvm) was chosen (Table 1). These values were selected based on the information that the $k_{L}a$ in frit spargers are 4–20 times higher when compared to drilled hole spargers at equal vvm.^44^ It is worth mentioning that the 0.75 L Multifors 2 system had an air cap that was set to 0.0033 vvm with a frit sparger (10 μm pore) and given the fact that the utilized sparger in the DASGIP system is an open pipe sparger, a range of 6–30 times more flow (in vvm) was explored. For the cultures realized at 40% DO, air caps of 1.5 L/H, 2.25 L/H, 3 L/H (0.038, 0.056, 0.075 vvm) were selected. For the 60% DO setpoint, the studied range for the air caps was 1, 3, and 5 L/H (0.025, 0.075, 0.12 vvm). Since impact of DO levels (60% and 40%) was observed, air caps that fit the 6–30× vvm criteria were explored at each DO setpoint. Feeding was increased for the 60% DO conditions as viability was observed to be extended at this setpoint. The new feed condition (abbreviated as F+ feeding regimen) was such that the total amount fed by the end of the culture (14 dpi) was a near 2‐fold increase (240 mL of feed for control process and 425 mL of feed for increased feeding). Instead of feeding a fixed amount in one rapid addition, the bioreactor receives a variable pre‐set amount depending on the current volume. The feed volume added with peristaltic pumps (at a slow flowrate) to the bioreactor is gradually distributed between sampling days. The fixed feed volume percentages were designed to follow the increase and plateau of a cell culture run to mimic dynamic cellular kinetics (as observed in Figure 1S). Given that osmolarity is known for CHO Feed 4 (range of 780–930 mOsm/kg) as well as BalanCD CHO Growth A basal medium (range of 290–310 mOsm/kg) and also feed volumes are known a priori, a 1.5‐2‐fold increase in Osm compared to the basal medium was expected with the F+ feeding regimen. To avoid instant excessive osmolarity increase (given that almost twice as much feed is added in the new feeding regimen F+ at 5, 7 and 10 dpi), a slow continuous feeding was implemented using peristaltic pumps such that the feed amount is distributed equally at slow flow rates between sampling days. Doing so the osmolarity increase is expected to be distributed across a longer time frame (days rather than minutes). Cultures were terminated at 14 dpi as this was determined to be the optimal duration. However, if viability fell below 50% before reaching 14 dpi, cultures were terminated early. Conversely, high‐performing cultures were extended beyond 14 dpi to assess viability beyond this period.

### 2.5 Bolus addition versus pump continuous feeding impact

In this study, air cap (1.5 L/H) and DO setpoint (60%) were kept constant across cultures while feeding dosage was altered (pump vs bolus) for two different strategies (feed volume calculated based on initial volume (FFIV) and based on current volume (FFCV), Table 1). A temperature downshift was realized on 3 dpi (6 days post‐seeding) on the cultures with increased feeding (F+) to evaluate if further gains on productivity or cell growth could be obtained by exposing the cells to optimal growth temperature for a longer period of time given the fact that the new feed regimen allowed for higher nutrient availability. In the literature, temperature shift impacts on growth have been extensively characterized and modeled.^45^

### 2.6 Feed regimen variation study

In this part of studies, the same DO setpoint (60%) and air cap (1.5 L/H) were used across cultures. Temperature downshift day was changed from 3 dpi (6 days post‐seeding) to 2 dpi (5 days post‐seeding) on the dynamically fed cultures as it was observed from capacitance measurements that the exponential growth phase seceded by 2 dpi (data not shown) thus it was postulated that by realizing the temperature shift one day earlier, further gains in recombinant protein production could be realized.^45^ Capacitance and oxygen transfer rate‐based feedings rely on the integration of the online signal that is then transformed to a cumulative feed amount. This cumulative feed is differentiated to obtain the feed volume that is to be added on a given day. The feed pump flow rate is then adjusted accordingly. The constant feed per cell (CFPC) strategy relies on estimating the total amount of viable cells within the bioreactor at any given sampling event and adding enough feed such that each cell receives the same volumetric amount of feed. The three strategies (CFPC, OTR, Capacitance) were dosed in a slow constant speed pump fashion. The manual control feed addition methodology for OTR and capacitance encompassed the integration of the signal, followed by multiplication with a predetermined constant. This constant facilitated the transformation of units from the integrated variable to cumulative volume of feed. Subsequently, the daily updated cumulative feed underwent differentiation with respect to time, yielding a volume earmarked for addition on a daily basis. The feed flow rate was adjusted based on this calculated volume, ensuring the dispensation of the designated volume within a 24‐h timeframe. In the CFPC feeding strategy, the feed flow rate was adjusted every sampling day to maintain the feed constant with respect to the number of cells at sampling day. This manual control strategy proved effective in the absence of a feedback control loop, showcasing a tailored approach to maintaining precise feed flow control. In the control process (experiment 16, Table 1) predetermined feed percentages (feed volume/Initial medium volume) are used to estimate the feed volume to add on a bolus fashion every sampling day.

## 3 RESULTS AND DISCUSSION

### 3.1 Impact of DO

**FIGURE 2:**
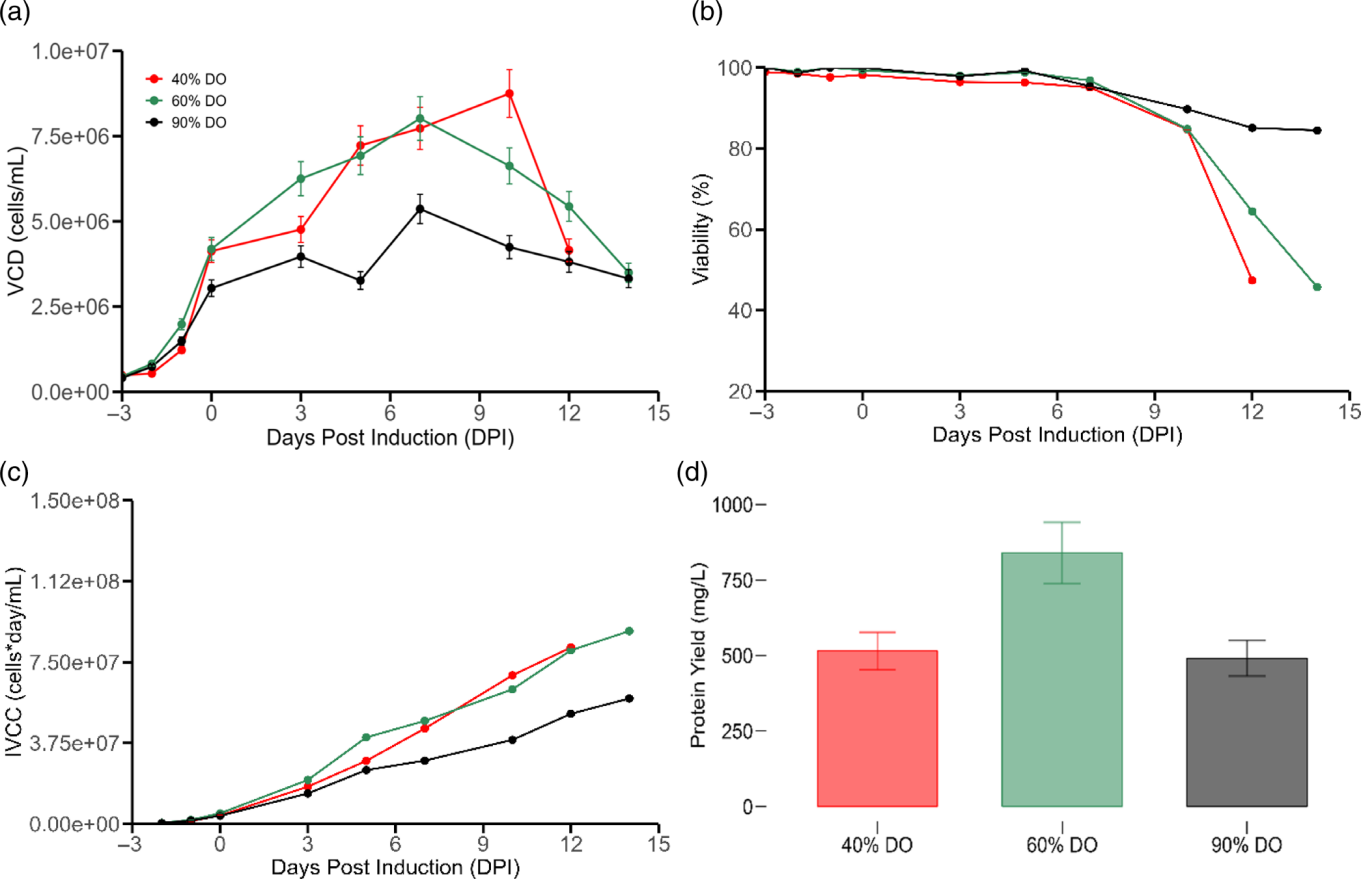
Impact of DO setpoint. (a) Viable cell density (b) viability (c) integral of viable cell concentration (IVCC) (d) end point titers. Red represents 40% DO, green represents 60% DO, and black represents 90% DO. Feeding initiation was concomitant with a temperature shift (from 37 to 32°C) and cumate induction occurred at 0 dpi. Error bars represent the measurement error associated to each variable utilizing a representative average relative standard deviation error.

As a first approach of evaluating adequate DO concentration, three different levels were explored: 40% DO, 60% DO, and 90% DO. In the literature, CHO cell cultures are normally cultivated at DO setpoints between 10% and 80% (% of air saturation).^46^ Prolonged exposure (10 or more days) to mildly hypoxic environments (20% DO setpoint) has elicited a similar hypoxic response as exposure of 1–3 days at 0.5%–5% DO.^47^ Therefore, 40% DO was chosen as the lower boundary to avoid mildly hypoxic environments that may be reached during DO control oscillations. Conversely, exposure to oxygen saturation levels above 200% DO has demonstrated alteration in the mitochondrial respiratory chain, lactate and alanine accumulation, and strong growth inhibition.^48^ DO levels above 100% can impact adversely growth and yield, and even minor changes from 50% to 75% DO were observed to significantly impact cell culture performance.^47^ Based on the aforementioned information, the upper bound was chosen to be 90% DO and a representative midpoint of 60% DO was studied. The DO levels were set with a 1.5 L/H air cap in order to determine the impact on viability, integral of viable cell concentration (IVCC), viable cell density (VCD), and end point titers. Figure 2a shows that 40% DO caused a sharper decline in viable cell density by 12 dpi when compared to both 60% and 90% DO. A similar trend was observed when repeating the 40% and 60% DO conditions with an air cap of 4.2 L/H (Annex Figure 2S). Importantly, DO control was tight avoiding overlap between conditions, ensuring that observable differences can be explained by DO levels (Annex Figure 3S). Figure 2b indicates that increasing the DO setpoint to 90% allowed to sustain high cellular viability over a longer time period. However, this did not translate into improvement in IVCC, as this condition had consistently low viable cell counts across culture time (Figure 2c). It must be noted that an optimal condition was found such that the final yield of 60% DO bioreactor had a significantly higher endpoint titer when compared to 40% and 90% DO cultures (Figure 2d). Thus, choosing an adequate DO level along with the appropriate air cap is paramount to get the desired process outcomes. Similar behavior has been observed in other cell lines as it is known that DO setpoints can impact specific respiration and specific production rates.^40^, ^49^ Moreover, it has been observed that brief exposure to reactive oxygen species (ROS) in the growth medium can impede the proliferation of CHO cells without triggering cell death, while encouraging intracellular maintenance.^50^, ^51^ As a consequence, it has been postulated that the accumulation of ROS in CHO cells might interrupt the exponential growth phase, as cells strive to counteract additional intracellular ROS buildup and avert cell death.^52^ This hypothesis is in line with the observation that the 90% DO setpoint decreased cell growth (Figure 2a). It has been proposed that specific respiration rates are closely linked to specific protein production rates^16^ which could explain why different protein yields were detected for the different DO levels. This link can be attributed to the fact that the metabolic activity of the cells has a direct impact on recombinant protein formation. Since the respiratory activity of mammalian cells correlates well to TCA fluxes, linear relationships between specific protein production and TCA cycle activity have been reported.^16^, ^17^, ^18^, ^19^, ^20^

### 3.2 Impact of aeration strategy and increased feeding volume

**FIGURE 3:**
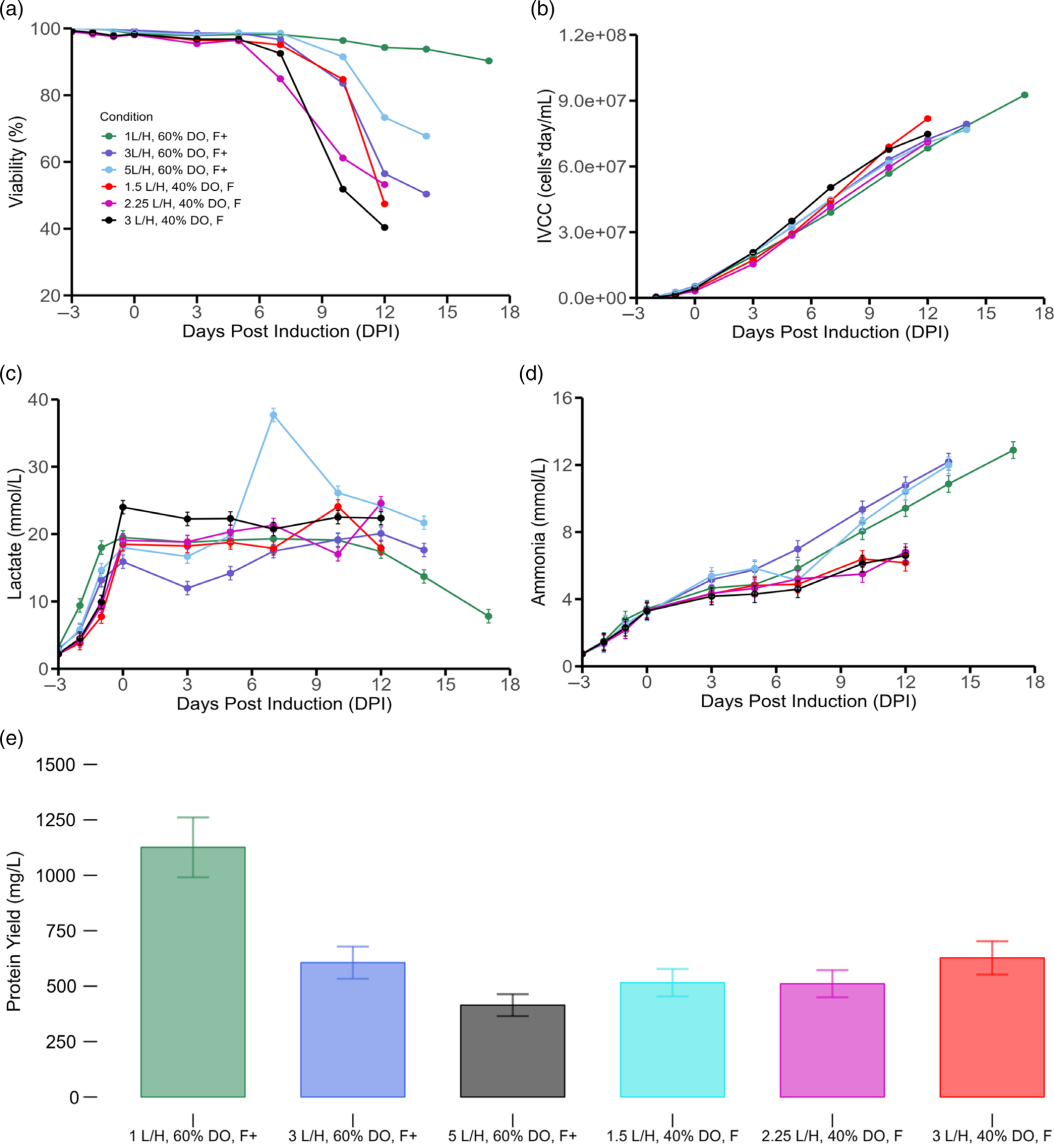
Impact of air caps and extra feeding. (a) Viability, (b) IVCC, (c) lactate accumulation, (d) ammonia profiles, (e) endpoint titers. Feeding initiation was concomitant with cumate induction and temperature shift (from 37 to 32°C) at 0 dpi for all the cultures. Feeding days correspond to 0, 3, 5, 7, 10, 12, 14 dpi. Error bars represent the measurement error associated to each variable utilizing a representative average relative standard deviation error.

For the cultures realized at a 40% DO level, increasing the air cap (1.5 L/H, 2.25 L/H, 3 L/H) had a negative impact on viability (Figure 3a). This impact on longevity also had an adverse impact on IVCC profile, given that the culture with the lowest air cap was able to reach higher cell concentrations (Figure 3b). This may be due, in part, to the fact that high aeration increases the amount of bubbles bursting at any given time, thereby augmenting the shear stress on the cells and thus adversely impacting culture performance^53^, ^54^ However, a high enough flow rate is needed to ensure sufficient ${\mathrm{CO}}_{2}$ stripping. Importantly, dissolved ${\mathrm{CO}}_{2}\left(d{\mathrm{CO}}_{2}\right)$ concentrations above 68 mmHg at bench scale^31^ have been observed to negatively impact protein productivity and cell growth. This observation is generally thought to be caused by the detrimental effects on internal pH and cellular metabolism that $d{\mathrm{CO}}_{2}$ accumulation has on mammalian cells.^55^ This effect can be thwarted by adding base into the system to keep pH constant. However, this by itself cannot be the only solution given that a stepwise increases in osmolality (caused by repeated base addition that counteracts $d{\mathrm{CO}}_{2}$ accumulation) can adversely influence cell culture outcomes as well.^56^ Additionally, high $d{\mathrm{CO}}_{2}$ concentrations have also been observed to negatively impact glycosylation profiles.^55^

As it can be seen in Figure 3a, at every air cap level, the combined 60% DO with gradually pumped feed (F+) outperformed the 40% DO with fixed feed (F) in terms of avoiding early cell culture termination. When looking at the IVCC profiles, again, the increased feed and increased DO cultures outperformed all cultures except the 1.5 L/H with 40% DO culture (Figure 3b). This was presumably due to the higher shear stress conditions associated with the 3 and 5 L/H cultures. Interestingly, we can see that the 60% DO condition with increased feeding and a 1 L/H air cap had a significant increase in end point viability (90% at 17 dpi) as well as a significant increase in IVCC (Figure 3a,b, respectively). When evaluating the corresponding lactate profiles, all the cultures with 40% DO levels and feed addition calculated based on initial volume (FFIV) reached a lactate production plateau and never achieved a consistent lactate consumption phase (Figure 3c). A similar conclusion can be drawn from the 60% DO with increased feeding in the two cultures with high air caps (3 and 5 L/H). Here, a lactate re‐production phase is initiated indicating high glycolytic activity. Interestingly, in the culture with low air cap, a consistent lactate absorption is discerned (Figure 3c). Once the plateau is reached, a slow decline until the end of the culture is observed indicating that the culture was able to enter a lactate net consumption phase. This air cap‐related impact to the lactate metabolism could be explained by the fact that at higher air caps, cells undergo greater environmental stress and thus have higher biosynthetic requirements for cellular reparation. Since one of the primary physiological functions of glucose is to provide the important building blocks for the biosynthesis of NADPH and nucleotides^20^, ^52^ and given the fact that NADPH plays a key role in the synthesis of macro molecules like fatty acids and amino acids,^57^, ^58^ higher glycolytic activities were usually observed with cells that underwent high shear stress conditions. A similar observation was realized in single‐use miniature bioreactors in which high levels of bubble damage caused declines in cellular viability that were concomitant with increased glucose utilization and lactate accumulation.^59^

When comparing the ammonia profiles, the increased pump feeding cultures (F+) have significantly more ammonia accumulation (Figure 3d). This is best elucidated by the fact that at 14 dpi, the concentration in the increased feed cultures is almost 2‐fold greater than the regular feed cultures. This may indicate that amino acid degradation took place because of greater amino acid consumption.^60^ This high level of ammonia did not seem to adversely impact the culture outcomes although considerations are required as product quality may be impacted.^61^, ^62^ However, in other pools or cell lines, such increases in ammonia could be harmful and as such, better feeding strategies that avoid unnecessary nutrient additions must be developed. When detailing the final titers, it is clear that the 60% DO with low air cap (1 L/H) and high feed condition (F+) was the best performing culture in terms of protein production (Figure 3e). It is also worth noting that this yield is higher than 60% DO with low air cap and standard feeding (F) indicating that there is an advantage towards increasing the overall feed amount. Moreover, for the increased volume (F+), more total recombinant protein can be extracted at the point of harvest when compared to the lower feed condition (F) thus representing an economic benefit. It must also be stressed that any advantage gained by having 60% DO and increased feeding (F+) is lost when subjecting the culture to higher air caps (Figure 3e). This suggests that setting an inadequate air cap can be detrimental to culture outcomes and, thus, finding proper aeration conditions must be performed in tandem with adequate feeding strategies. Since no added benefit in terms of protein production was observed between 14 and 17 dpi, subsequent cultures were terminated at 14 dpi.

### 3.3 Bolus feed addition versus slow continuous pump feeding strategy

**FIGURE 4:**
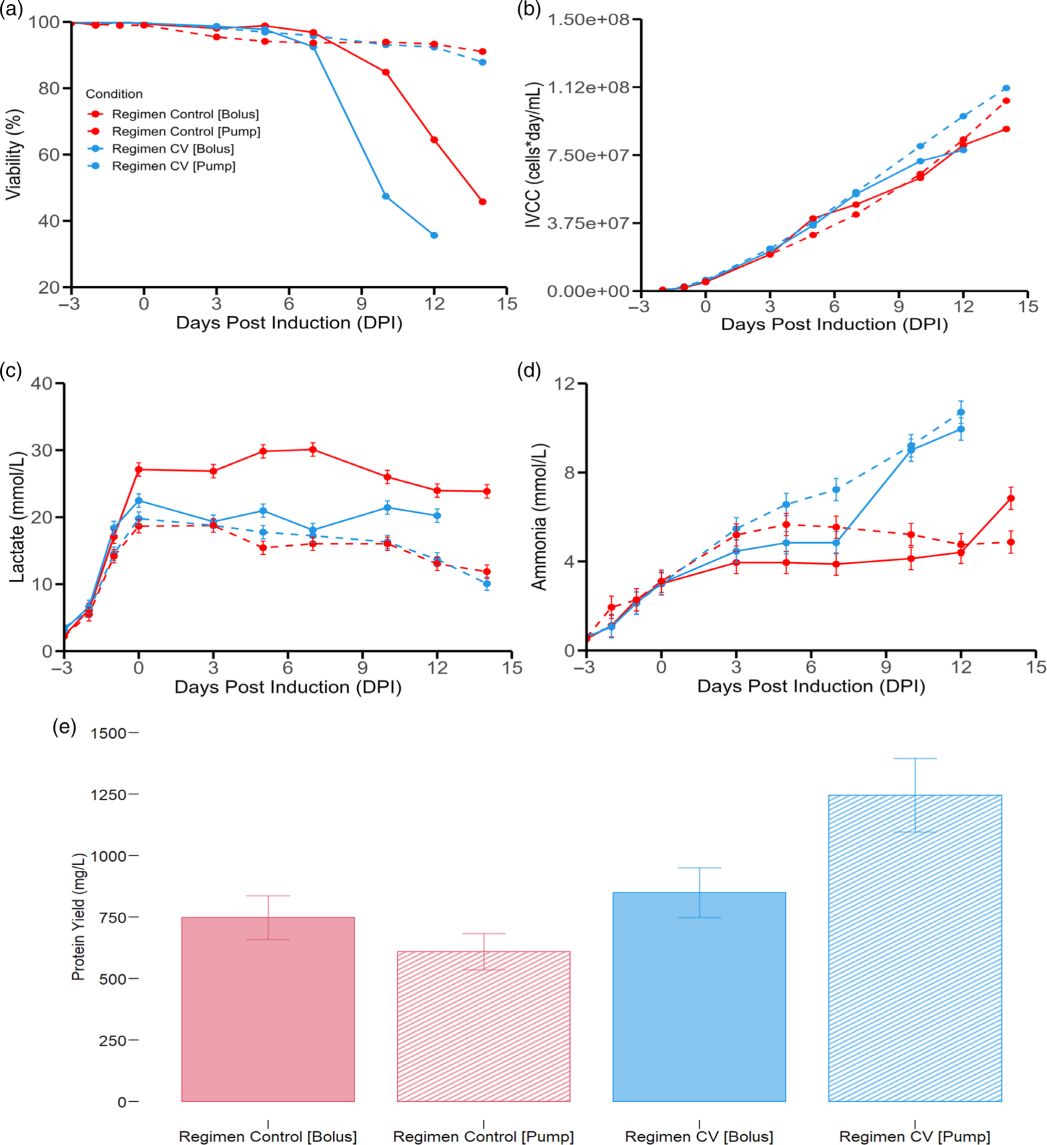
Pump versus bolus feeding impact. (a) Viability, (b) IVCC, (c) lactate accumulation, (d) Ammonia profiles, (e) End point titers. Solid red represents Regimen Control [Bolus]; Dashed red represents Regimen Control [Pump]; Solid blue represents Regimen CV [Bolus] and dashed blue represents Regimen CV [Pump]. Feeding initiation was concomitant with cumate induction at 0 dpi. A temperature shift (from 37 to 32°C) was realized at 0 dpi for Regimen CV [Bolus] and Regimen Control [Bolus] while the temperature shift was realized at 3 dpi for Regimen Control [Pump] and Regimen CV [Pump]. Error bars represent the measurement error associated to each variable utilizing a representative average relative standard deviation error.

An experiment comparing feed addition based on bolus dosing to slow pump dosing was devised to evaluate the impact of slow feed addition as it was not immediately clear if the increased feeding alone was responsible for the increased performance or if it was the interaction between the increased feeding and the slow feed addition process. A change in temperature shift for the slow pump feed condition was realized to determine if substantial gains could be made with the onset of nutrient addition. For all conditions, 60% DO was used with a 1.5 L/H air cap as low air caps were confirmed to be the best performing. As it can be visualized in the cultures with slow pump addition, regardless of the total amount of feed given (control feed calculated based on initial volume (control) and feed calculated based on current volume (CV)), both cultures had significantly higher viability by 14 dpi (Figure 4a). It must be stressed that the feeding volume based on current volume, by 14 dpi, resulted in 1.85‐fold increase of the total feed volume added when compared to the feeding based on initial volume. Interestingly, the culture with increased feed (based on current volume) that was dosed in a bolus style had to be terminated early given that it suffered an early culture crash that was concomitant with a high bolus addition (110 mL) realized at 7 dpi (Figure 4a). The benefit of slow pump feeding is that it allows to increase the amount of feed without generating adverse culture outcomes. Presumably, this culture crash is related to rapid increase in nutrient concentrations (glucose and amino acids) that, in turn, causes an abrupt change in osmolarity. This sudden osmolarity increase can be tolerated by the tested CHO pool within a threshold. The addition of 32.5 mL (between 0 and 5 dpi) by bolus did not immediately impact cell viability (control feed regimen) while the addition of 48.5 mL at 7 dpi (control feed) did result in a decrease in viability when measured at the next sampling point (Figure 4a). Importantly, when 110 mL was fed (regimen based on current volume, CV) in a bolus fashion at 7 dpi, an even steeper decrease in viability was noted, thus demonstrating a step‐wise decrease in viability given that ever‐increasing amounts of feed (given in a bolus way) resulted in incrementally worse culture outcomes (Figure 4a). This observation may be due to various reasons. It could be due to a rapid increase in volume, critical decrease in nutrient concentrations between sampling days or variations in osmolarity. Since volume changes of 100 mL were not observed to drastically decrease 𝑘_𝐿_𝑎 (𝑘_𝐿_𝑎 for 650 mL at 1.5 L/H air cap is 2.16 while the 𝑘_𝐿_𝑎 for 750 mL at 1.5 L/H air cap is 1.96), and oxygen supplementation is anyway added as needed so as to maintain the DO constant, no direct impact of oxygen limitation on cellular viability can be expected. Nutrient concentrations were not observed to be critically low when comparing sampling days as glucose concentration was always controlled to be above 17 mmol/L (3 g/L). Amino acid concentrations are not depleted during the production phase as seen in annex Figure 4S. When observing osmolarity measurements, it was noted that at 7 dpi, the osmolarity levels were equivalent for regimen CV pump and regimen CV bolus (310 mOsm/kg for pump and 313 mOsm/kg for bolus). However, by 10 dpi, regimen CV bolus exhibited a spike in osmolarity to 427 mOsm/kg while regimen CV pump registered a significantly lower value of 331 mOsm/kg. Given that both conditions were subjected to identical feeding and thus identical increases in nutrient concentrations by each sampling day, it is postulated that the observed increase in osmolarity results from decreased metabolic activity caused by cellular death. This decreased activity leads to decreased nutrient consumption and thus increased accumulation of nutrients in the media driving an increase in observed osmolarity. This is best exemplified by the differences in glucose consumption per day. For both regimen CV pump and CV bolus, glucose consumption per day is very similar (6.28 and 6.63 mmol/L*day respectively) at 7 dpi. However, by 10 dpi, the glucose consumption per day begins to diverge such that for regimen CV pump the value is 7.81 mmol/L*day while for regimen CV bolus it is 5.2 mmol/L*day. This trend continues onto 12 dpi where glucose consumption remains high for regimen CV pump (5.92 mmol/L*day) and for regimen CV bolus, a large decrease in glucose consumption per day is detected (2.19 mmol/L*day). This would explain the substantial difference in osmolarity measurements after 10 dpi where a large viability crash is detected. Consequently, it can be postulated that rapid osmolarity increase is a key factor driving the sudden decrease in cellular longevity. Since both cultures were fed the same total amount across sampling days, it stands to reason that the driver of cellular death is the difference in time in which the cultures were exposed to the increase in osmolarity. For the bolus culture, this osmolarity increase took place in the time frame of 1 h or less, while for the pump fed cultures, the time frame is 48 h or more. Similarly, for regimen control, osmolarity is nearly identical at 5 dpi (342 mOsm/kg for pump and 340 mOsm/kg for bolus) but begins to diverge by 7 dpi (344 mOsm/kg for pump and 387 mOsm/kg for bolus). By 12 dpi, osmolarity in control regimen pump is 356 mOsm/kg while for control regimen bolus it is 417 mOsm/kg. Concomitant with this change in osmolarity is a change in viability outcomes (93% for pump and 64% for bolus). Critically, proving the hypothesis that rapid variations in osmolarity are more negatively impactful to culture longevity when compared to slow increases in osmolarity (change in osmolarity spreads over several days) requires more frequent measurements. Such approach can be undertaken with conductivity measurements as positive correlations between osmolarity and conductivity has been found in the literature.^63^, ^64^ As can be seen in Annex Figure 5S, conductivity measurements for bolus feed addition and pump feed addition show distinct patterns of stepwise increase (bolus feed addition) versus a slow linear increase (pump feed addition). Consequently, the observed difference of viability outcomes between bolus addition and pump addition may be explained by the fact that sudden bolus feed additions lead to rapid increase in conductance (and thus osmolarity), while slow pump addition generates a slow increase in conductivity measurements, and by extension, a slow increase in osmolarity that may allow the cells to adapt to the changing environment throughout the process. In the literature, it has been noted that hyperosmotic stress can drive cell death through necrosis and apoptosis.^65^, ^66^ Furthermore, it has been observed that CHO‐S cells can be adapted to hyperosmotic conditions (>450 mOsm/kg) by repeated passaging (more than 10 times) while still remaining unaffected in terms of overall cell growth.^67^ This approach was suggested to allow CHO cells to avoid being affected by rapid osmolarity increases caused by large bolus feed additions.^67^

When evaluating the IVCC profile of the cultures, one can see that the increased feed with slow pump addition outperforms all the cultures (Figure 4b). This is due to the fact that viability and high cellular densities are maintained over the whole process. Lactate profiles show that bolus additions do not facilitate a transition from lactate production to lactate consumption phase (Figure 4c). In fact, gradual increases and decreases in lactate concentrations are associated with every feeding event, indicating that the sudden bolus additions alter transiently the metabolic behavior of the cultures (Figure 4c). In contrast, the slow pump additions show a slow decrease in lactate concentration throughout the culture run. This could be explained by the fact that without the sudden changes in glucose concentrations, no sudden glycolytic influx is activated, and the culture is allowed to sustain a lactate absorption phase. This is important given that lactate absorption has been observed to be a key process indicator (KPI) for protein production and thus it is desirable behavior.^68^ When evaluating ammonia concentration, it was shown that the increased feeding strategy (pump and bolus) leads to higher ammonia accumulation as previously observed (Figure 4d). This was detected regardless of dosage method (slow pumping or bolus addition). Despite the increase in ammonia accumulation, it cannot be implied that said outcome negatively impacted IVCC, longevity or yield. When discerning the final titer concentration, it is clear that increased feeding yields superior results, but only if it is dosed through slow pump addition (Figure 4e). Here, the sudden changes in nutrient concentrations and osmolality variations are avoided. In the literature, slow feeding strategies have been used with CHO‐K1 cell lines for the production of monoclonal antibodies.^69^ It was determined that when compared to bolus feeding methods, slow pump feeding did not show any advantage when the feed amount was low. However, with high feed amounts, the pump method allowed for the reduction of metabolic‐by‐product buildup.^69^ A similar strategy was utilized in large‐scale expansion of mammalian cell spheroids. It was shown that slow pump feeding eliminated fluctuations in nutrients levels and consequently improved cell growth.^70^ Similar observations were realized in a CHO‐S cell line where pump feed addition outperformed bolus feed addition in terms of IVCC and IgG production. However, viability was unaffected between both conditions.^67^ A pulsating feeding strategy has also been applied to glucose and amino acid supplementation resulting in enhanced mAb production and increased endpoint viability.^71^ Since these results show that slow pump feeding is applicable to CHO stable pools producing SARS‐CoV‐2 spike protein, it could be suggested that the advantages of slow feeding hold across a wide range of mammalian pools and cell lines and should be systematically assessed. The delayed temperature shift was observed to have a positive impact on IVCC profiles (Figure 4b vs Figure 3b) regardless of feeding strategy (based on Initial volume or based on Current volume). However increased yields were only observed in the cultures with increased feeding. Given that slow pump feed addition allowed for more aggressive total feed supplementation, it is possible that future gains with respect to specific protein production can be obtained since it has been observed that hyperosmotic stress on CHO cells can enhance specific productivity.^72^ Indeed, this phenomenon has also been observed in hybridoma cultures.^73^, ^74^

**FIGURE 5:**
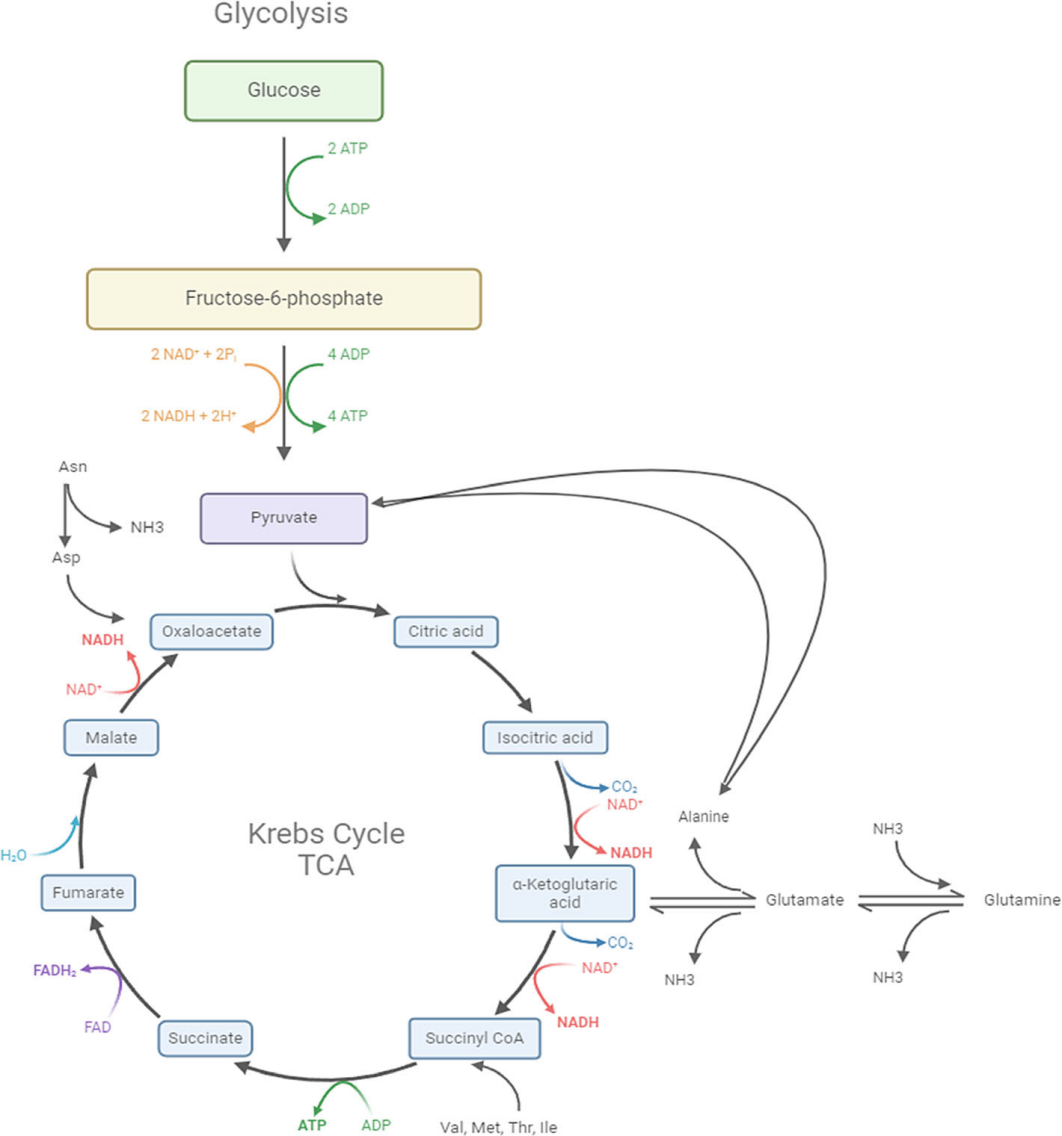
Schematic of mammalian cell metabolism: Glycolysis and TCA cycle.

When detailing the amino acid concentrations in Figure 4S, it can be discerned that His, Ser, Arg, Gly, Glu, Pro, Thr, Cys, Met, Ile, and Phe concentrations begin accumulating at greater amounts by 10 and 12 dpi in the CV Bolus condition when compared to the CV Pump condition. His, Ser, Arg, Thr, Cys, Met, Phe, Ile, Leu and Val are known to be consumed during the production phase and at the beginning of the decline phase in CHO cell cultures.^17^, ^75^ A simplified schematic of mammalian cell metabolism and the relationship between glycolysis and the TCA cycle can be viewed in Figure 5. An increase in the concentration of these amino acids in the CV Bolus was observed when compared to CV Pump. It makes sense as by 10 dpi, CV Bolus viability drops below 60% thereby diminishing the number of cells consuming the available amino acids in the feed and basal media.

Glutamate is added to the feed so as to aid in glutamine synthesis for CHO‐GS cell lines.^76^ Its accumulation in the medium could indicate that glutamine synthesis is decreasing and consequently its metabolic activity is waning. Given that by 10 dpi the viability of CV Bolus drops below 60%, its reduced metabolic activity led to greater accumulation of glutamate when compared to CV Pump. Furthermore, CHO cells face challenges in synthesizing an adequate amount of proline to support essential cellular processes such as de novo protein synthesis and consequently proline is supplied via feed supplements.^77^ As such, differential accumulation between Bolus and Pump conditions may indicate inadequate metabolic activity in cellular maintenance and recombinant protein production which can be explained by the 60% viability drop by 10 dpi in the CV Bolus condition. Glycine is a product of serine metabolism which fuels one‐carbon (1C) units metabolism.^78^ This amino acid is present in low concentrations in both the feed supplied (0.67 mmol/L) and in the basal medial (1.41 mmol/L) and is involved in the synthesis of glutathione.^60^ Since there have been hints at a potential connection between glutathione (GSH) levels and cellular productivity of recombinant proteins,^79^, ^80^, ^81^ it stands to reason that increased accumulation of glycine in the CV Bolus condition may be interpreted as lower GSH synthesis activity caused by the viability crash which led to a decrease in the oxidative metabolism that is generally associated with high protein production.^18^ This is evident by the fact that peak pure oxygen flow in the CV Pump condition was 3.3 L/H at 12 dpi while for the CV Bolus condition it was 2.2 L/H at 6 dpi.

**FIGURE 6:**
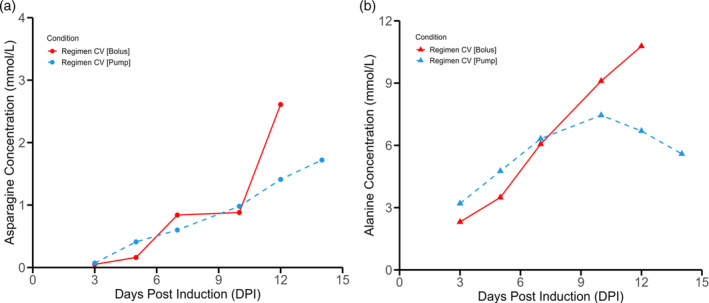
CV[Pump] and CV[Bolus] feeding regimen impact on asparagine and alanine profiles. (a) Asparagine (Asn) concentration in the spent media, (b) Alanine (Ala) concentration in the spent media. Feeding initiation was concomitant with cumate induction at 0 dpi. A temperature shift (from 37 to 32°C) was realized at 0 dpi for Regimen CV [Bolus] while the temperature shift was realized at 3 dpi for Regimen CV [Pump].

In Figure 6, it is possible to observe the concentration profiles for asparagine (Figure 6a) and alanine (Figure 6b). During the plateau phase, asparagine represents about 8% of incoming carbon source.^18^ In fact, it has been determined that multiple TCA cycle intermediates (e.g., citrate, malate, succinate) derive substantial carbon from asparagine catabolism.^82^ It has been delimited as a key nutrient that has to be replenished to avoid rapid depletion.^83^, ^84^ A recent CHO metabolism review paper has noted that asparagine is significantly consumed during the production phase and the decline phase.^17^ Therefore, it makes sense to observe differential accumulation in the media for the Regimen CV Bolus and Regimen CV Pump conditions. As the large bolus addition of feed is added to the CV Bolus condition at 7 dpi, the subsequent viability crash diminishes metabolic activity and consequently asparagine uptake requirements. Interestingly, the alanine profile (Figure 6b) is observed to diverge between CV Bolus and CV Pump conditions by 10 dpi. Generally, alanine is observed to accumulate in the extracellular media both in production phase and the decline phase.^17^, ^75^, ^85^ There is no alanine in the feed formulation and a low amount in the basal media (0.53 mmol/L). It is hypothesized that the secretion of alanine into the cell culture serves as a strategy to mitigate ammonium toxicity by functioning as a reservoir for nitrogen.^86^ It is thought that alanine biosynthesis serves as a stress response mechanism for metabolic waste accumulation.^87^ Additionally, the metabolic interconnection between glutamine and alanine involves the glutamine‐pyruvate transaminase reaction, wherein glutamine is transformed into α‐ketoglutarate, and the resulting amine group is transferred to pyruvate to produce alanine.^88^ These transaminase reactions, including those mediated by glutamine‐pyruvate and glutamate–aspartate transaminase, allow CHO cells to mitigate excess ammonia production, potentially contributing to the establishment of a more favorable cellular environment in culture.^88^ Interestingly, in CHO cells, alanine concentrations have been observed to diminish during the production phase of a culture and as such it has been suggested that alanine is important in metabolic processes related to recombinant protein synthesis.^89^ Tangentially, excessive alanine accumulation is known to be a negative in CHO cultures, as it can serve as an allosteric inhibitor of pyruvate kinase by signaling an abundance of intermediates from the tricarboxylic acid (TCA), thereby inhibiting pyruvate kinase and the TCA pathway,^90^ this can be best visualized in Figure 5. It may be interpreted that alanine consumption observed in CV Pump condition underscores increased TCA cycle activity which can be further underlined by the higher oxygen (3.3 L/H > 2.2 L/H) requirements and protein production (1246 mg/L > 850 mg/L) when compared to the CV Bolus condition.

No clear impact regarding delayed temperature shift for regimen CV pump when compared to regimen CV bolus can be discerned. The main driver of difference can be attributed to the difference in dosage method as the difference in dosage method explains the difference in longevity and metabolic activity. For illustration, when comparing regimen CV pump to the previous set of experiments (1 L/H air cap, 60% DO with regimen CV pump, temperature shifted at 0 dpi), marginal gains in titer at 14 dpi (1193 mg/L vs 1246 mg/L) and increased IVCC at 14 dpi (7.85 × 10^7^ cells*day/mL vs 1.12 × 10^8^ cells*day/mL) are detected. Here, the delayed temperature shift can be observed to allow for more biomass accumulation and a marginal improvement in titer. Despite the temperature shift difference between these two conditions, longevity remains high at 14 dpi (viability is around 90%) and metabolic activity is also comparable at 14 dpi (glucose consumption of 6.02 mmol/L*day for the regimen CV pump culture and 5.33 mmol/L*day for the 1 L/H air cap, 60% DO with regimen CV pump, temperature shifted at 0 dpi culture).

### 3.4 Dynamic feeding strategies

Given that slow pump feeding was observed to be the optimal dosage, various dynamic feeding strategies were devised and assessed to optimize the process. These feeding strategies were set up to respond to biological signals rather than feed based on a set amount that was determined a priori. Constant feed per cell based on manual cell counts (CFPC), feeding based on capacitance signal (CAP), feeding based on OTR signal were compared to the standard process of feeding that relies on bolus additions calculated based on initial volume. For the three strategies, the onset of feeding was one day before induction (−1 dpi) since this was observed to be a key moment given that the cultures reach air cap at this point and thus require additional oxygen supplementation (Figure 6S in Annex contains a typical DO profile with online air cap and $O_{2}$ flow). This may indicate that the cultures are very metabolically active (probably through glycolysis path to rapidly produce 2 ATP per mole of glucose to support rapid cell growth), thus the cells are in need of additional nutrients. For the CAP and OTR feed regimens, a daily adjustment of the feed flow rate to align with the desired signal trajectory was realized while for the control and CFPC regimens feed additions are realized every sampling day.

**FIGURE 7:**
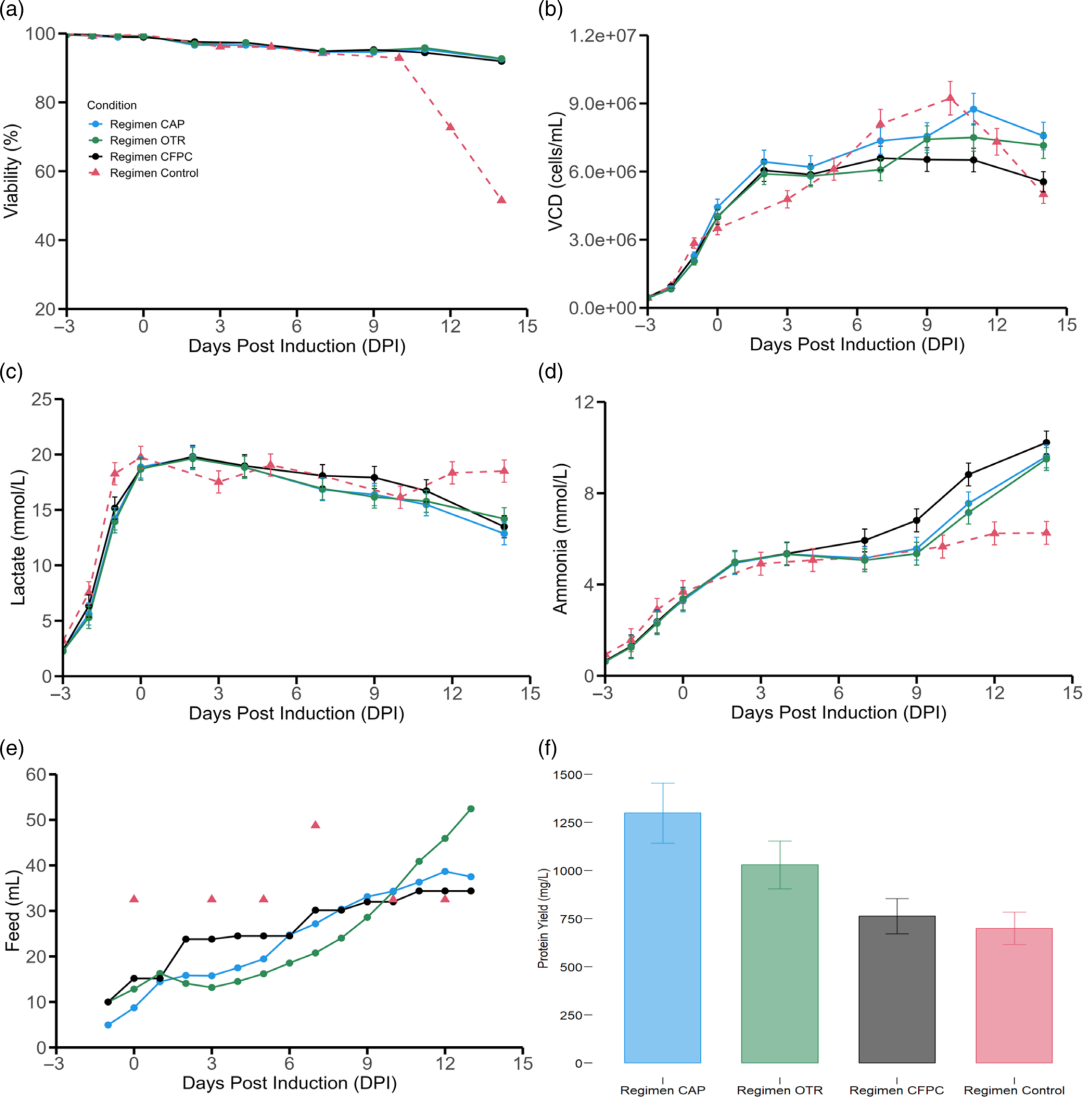
Impact of biologically relevant feeding strategies. (a) Viability, (b) viable cell densities, (c) lactate accumulation, (d) ammonia accumulation, (e) daily feed volume additions, (f) endpoint titers. Initiation of feeding was concomitant with temperature shift and cumate induction at 0 dpi for the control regimen. For regimen OTR, CAP and CFPC, feeding was initiated at −1 dpi, cumate induction was realized at 0 dpi and temperature shift was realized at 2 dpi. Feeding days correspond to 0, 3, 5, 7, 10, 12 dpi for the control regimen and −1, 0, 1, 2, 3, 4, 5, 6, 7, 8, 9, 10, 11, 12 dpi for regimens CAP, OTR and CFPC. Error bars represent the measurement error associated to each variable utilizing a representative average relative standard deviation error.

**FIGURE 8:**
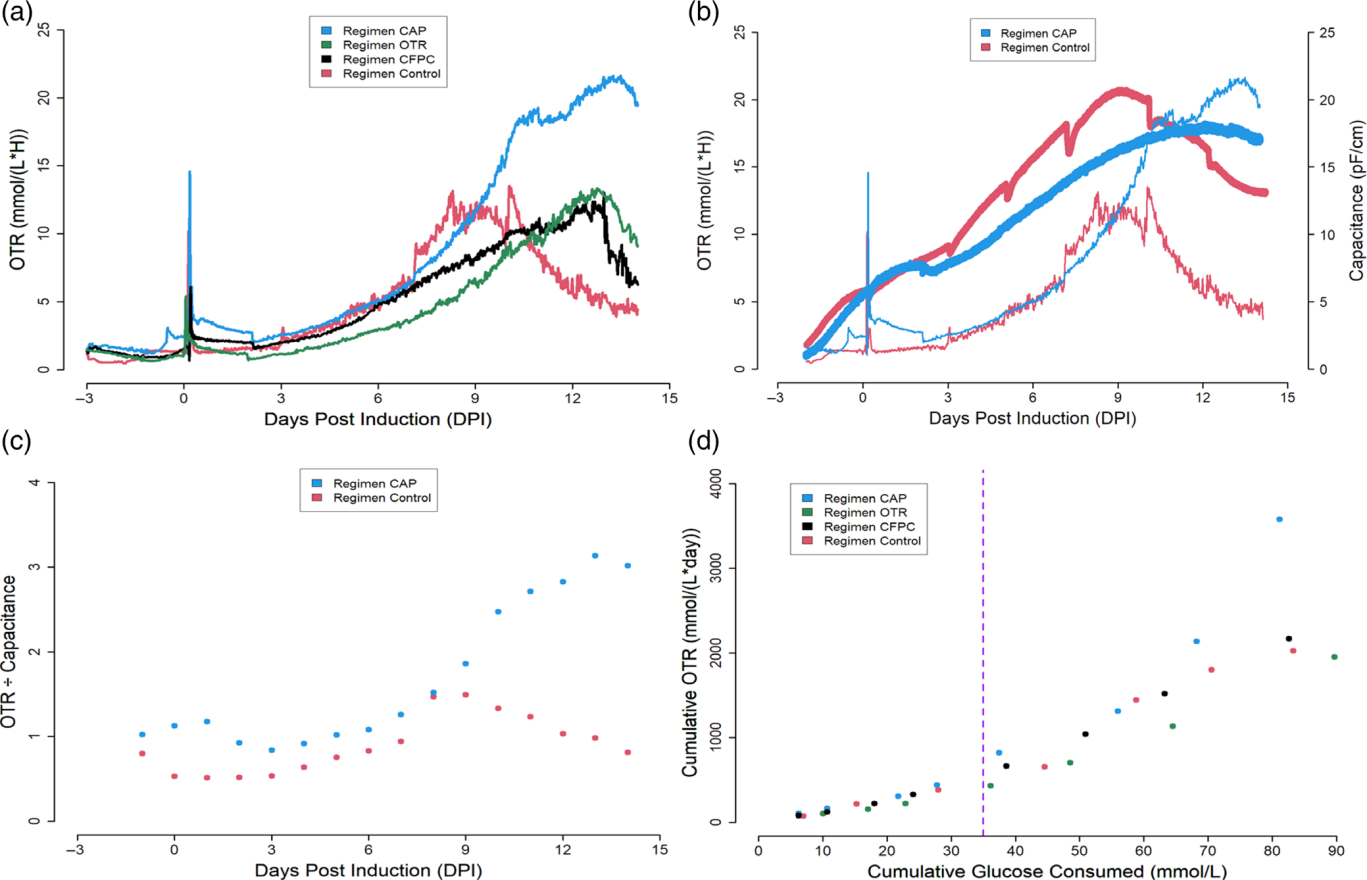
OTR and bio‐capacitance evolution for the various feeding strategies. (a) OTR measurements for the various feeding strategies. (b) OTR and bio‐capacitance signal overlay. Thin lines (red and blue) are the OTR signals while the thick lines (red and blue) are bio‐capacitance signals. (c) Daily specific volumetric respiration rates (ratio of daily OTR measurements and daily capacitance measurements). (d) Cumulative OTR versus cumulative glucose consumption scatter plot. Purple line separates 4 dpi from 7 dpi datapoints.

For all three dynamic feeding strategies, culture longevity was maintained longer when compared to the standard process probably due to the dosage or the varying amount or a combination of both effects (Figure 7a). It is also clear from the viable cell density plot (Figure 7b) that after the temperature downshift at 2 dpi, cell concentrations for the CFPC, CAP, OTR conditions were maintained for longer periods of time. Importantly, for the three dynamic feeding strategies, cell concentrations remain high after temperature shift while the control process continues growing after temperature shift, reaches a peak VCD value and subsequently crashes. Lactate again can be observed to enter a consumption phase soon after 2 dpi which was concomitant with the temperature downshift (Figure 7c). Ammonia build‐up, even though higher than in the standard process, is overall less when compared to the previous regimen with feed volume calculated based on current volume. This could be an indication that, since the feeding strategies relied on biologically relevant signals, the degradation of amino acids in the media due to over feeding was diminished (Figure 7d). From the daily feed volume profile, one can see how the strategies were able to trace the relevant signal (Figure 7e). While CFPC relies on viable cell concentrations (Figure 7b), the feed based on capacitance signal is able to track the time evolution profile of the permittivity signal (Figure 8b) and the OTR‐based feeding strategy is enabled based on the changes in OTR (Figure 8a). As it can be discerned from the endpoint titers (Figure 7f), the three dynamic feeding strategies outperformed the standard process such that capacitance‐based feeding (CAP) > OTR based feeding > cell count based feeding (CFPC) > bolus feeding based on a fixed amount related to the initial volume. This ranking could be linked to the fact that both bio‐capacitance and OTR‐based feeding strategies are indirectly monitoring biovolume and its consequent metabolic activity while the cell count based feeding is assuming that each cell should receive the same amount of feed per day. Thus, conceptually speaking, the two feeding strategies (CAP and OTR‐based) are much more dynamic in terms of considering changes in metabolism or total biovolume. Regimen CFPC had a lot more feed addition between 2 and 6 dpi when compared to the best performing regimens (CAP and OTR). This sub optimal addition at the start of the production phase may have contributed to worse protein production given the fact that CFPC has lower endpoint protein production (762 mg/L) when compared to CAP (1300 mg/L) and OTR (1029 mg/L). It must be noted that when comparing the protein yield of regimen CV pump (1250 mg/L) to regimen CAP and OTR similar high performance is observed. Given that the feed profile of regimen CV pump was pre‐set to track expected changes in cell counts during the cultivation process (Figure 1S) thus serving as a proto‐dynamic feed regimen strategy. It may be hypothesized that the changing feed profile along with slow continuous addition of feed is one of the mayor explicatory reasons for the high protein yield results in regimen OTR, CAP and CV pump.

From the oxygen uptake rate plots (OTR = OUR due to a constant DO setpoint maintained in the bioreactors) (Figure 8a), it is clear that feeding based on capacitance signals induced higher overall oxygen requirements which are in part explained by a higher VCD that was sustained throughout the culture process. Additionally, the increased OUR may be linked with an increased protein expression as this culture was able to outperform all the tested feeding strategies (Figure 7f). In the cultures that were subjected to a 2‐dpi temperature downshift (CFPC, CAP, OTR), a temporary decrease in OUR is observed from 2 to 4 dpi indicative of a decreased metabolic activity probably due the decrease of temperature from 37 to 32°C (a concomitant decrease in glucose consumed per day was also noted as shown in Annex Figure 7S). This has been noted in the literature as decreases in temperature impact specific respiration rates.^49^ It has been found that mild hypothermia when coupled with nutrient supplementation can increase specific protein production due to its inverse relationship with the fraction of cells in S phase during the cell cycle.^91^ When overlaying the resulting OUR and capacitance signals, several points of interest can be noted. First, peak bio‐capacitance and peak oxygen consumption measurements happen around the same points in time (7–10 dpi for the control regimen and 10–14 dpi for the CAP regimen), indicating that both oxygen consumption and bio‐capacitance are strongly related to viable cell volume as has been suggested in the literature.^19^, ^26^, ^92^, ^93^, ^94^ Second, decrease in oxygen consumption precedes cell death and it is steeper than the decline in cell counts (Figure 8b). This could be due to the fact that once the cells are close to the decline phase, their metabolic activity slows down considerably. This is evident from the reduction in glucose consumed per day for the regimen control condition while daily glucose consumption rate remains high in the CAP condition (Annex Figure 7S). Third, the increase of the bio‐capacitance signal (from 6 to 18 pF/cm) between 2 and 12 dpi (Figure 8b) cannot be explained alone by the secondary increase in cell counts (from 6.5 × 10^6^ to 9 × 10^6^) after induction (Figure 7b). This increase in cell counts was also accompanied by an increase in cell size. Historical records for this cell pool show radius increased from 14.9 μm at inoculation (−3 dpi) to 16.8 μm at 12 dpi. A similar observation was realized on a CHO cell line where the cellular diameter was increased from 13.6 μm before induction to 15.5 μm 9 days after induction.^95^ For illustration, a 2 μm increase in radius represents a 1.43‐fold increase in cellular volume.^8^, ^9^, ^10^ Fourth, both OUR and bio‐capacitance signals show similar trends for the bolus feeding process. Here both signals grow almost exponentially between 3 and 9 dpi, which is in response to strong secondary growth phenomenon. In the pump fed cultures, the bio‐capacitance and OUR curves also show similar behaviors. The capacitance signal shows a convex trend while the OUR signal displays a concave profile. This may in part be due to the fact that strong secondary growth is not observed. Since there is no strong secondary growth in this case, the increase in capacitance must necessarily be due to increase in cellular size while the OUR increase is due to both an increase in cell size and an increase in metabolic activity. Nonetheless, both signals have an increasing trend thanks to the capacity of the signals to directly (capacitance) or indirectly (OUR) detect biovolume which can also serve as a proxy for metabolic requirements (bigger cells have higher oxygen requirements). As it can be seen from the titer production profiles (Annex Figure 8S), a correlation is observed such that when the cultures are ordered in terms of peak oxygen requirements at the end of the culture process, they are consequently also ordered in terms of peak protein expression. This could be possibly explained by the fact that close relationships between recombinant protein expression and enhanced TCA cycle activity have been observed.^20^

Interestingly, when evaluating the biovolume specific respiration rates (OTR/Capacitance) for the bolus‐fed process and the continuously fed capacitance‐based process, it is clear that biovolume specific respiration rates are higher in the slow pump process when compared to the bolus‐fed process (Figure 8c). Given the strong link between specific protein expression rates and specific respiration rates, it can be postulated that this is a key reason as to why higher protein titers were reached in the CAP regimen.

When comparing the cumulative OUR versus the cumulative glucose consumption plot after 4–5 dpi, there exists a change in the relationship between oxygen requirements and glucose utilization (Figure 8d). In essence, the total oxygen consumed increases at a higher rate (increasing slope) when compared to the increase in total glucose consumption. Since lactate begins to decrease after 4 dpi and protein expression begins to increase dramatically between 4 and 12 dpi, it can be postulated that these two key nutrients (glucose and oxygen) are being fluxed into the TCA cycle to be used as part of protein synthesis. Metabolic shifts can also be monitored such that a change from highly glycolytic metabolism to predominantly oxidative metabolism can be detailed. Given the positive correlation that has been observed between oxidative metabolism and protein production, it is a parameter to consider when analyzing OUR data. Thus, estimating OUR and bio‐capacitance in real time and contrasting it with measured metabolic rates can serve as a basis for soft sensing changes in metabolism that in turn are related to process outcomes like levels of protein expression.

## 4 CONCLUSION

In the scope of this article, improvement of culture outcomes in terms of longevity, IVCC and SARS‐CoV‐2 spike protein production was achieved through various aeration and feeding strategies. Firstly, it was observed that appropriate DO setpoints had important effects in terms of increasing endpoint viability and protein production yield. At the same time, it was determined that setting the appropriate sparging air caps also has a significant impact on cell culture kinetics such that adverse conditions (e.g., high air caps) impact not only cell culture longevity but also lactate accumulation profile. Presumably, this is a consequence of sub‐optimal environmental hydrodynamics which alters the metabolic pathways of the cells. Although not shown within this article, low air caps can also be detrimental given that insufficient carbon dioxide stripping can be observed especially when scaling the process. Thus, finding the adequate aeration conditions is paramount for a scalable process. It was also determined that increased feeding was only beneficial when coupled with slow pump addition as it diminished the cellular stress perhaps due to attenuating nutrient concentrations oscillations and exposing the cells to increased osmolarity at very low feed addition rates. Thus, it can be said that feed amount and dosage method are important components of feeding strategy development. This dosage strategy (slow pump) was also observed to allow for consistent lactate consumption which is known to be a good process indicator in CHO cells. It was also determined that dynamic feeding strategies (oxygen uptake rate and bio‐capacitance based) can increase titer when compared to the control process. Given the increase of monitoring technologies in the bio‐therapeutical industry, it is then appropriate to begin designing strategies that react directly to measured online signals as it can be easily automated using feedback control loops, thus diminishing the day‐to‐day workload that operators have. The suggested signals are bio‐capacitance and OTR (if accurate DO control is achieved) given their close links to viable cell volume (biovolume) rather than cell counts and consequently their indirect relationships to increases in metabolic demands (larger cells have been observed to have increased respiratory demands). Alternatively, if no accurate DO control can be achieved, OUR estimation can be realized through standard respiration tests in which cyclical measurement of the DO extinction profile is realized when air supply is stopped.^23^ An additional take‐away from this research is that feeding strategies and processing parameters must be optimized in tandem since the positive impact of dosage and increased feeding can be undone by adverse aeration conditions and inappropriate DO setpoints. Future work can center around evaluating process related impacts on quality profiles of the SARS‐CoV‐2 spike protein (glycan monosaccharide analysis, human ACE2 affinity, trimerization status and purity, identification of the N‐ and C‐terminal and thermal stability). Importantly, product quality attributes for this cell pool have been shown to be highly robust and comparable to cell line expression methods.^3^

## AUTHOR CONTRIBUTIONS

**Sebastian‐Juan Reyes:** Investigation; conceptualization; methodology; data curation; writing – original draft; formal analysis; writing – review and editing. **Phuong Lan Pham:** Writing – review and editing; methodology; formal analysis; supervision; funding acquisition. **Yves Durocher:** Writing – review and editing; supervision; funding acquisition; resources; formal analysis; project administration. **Olivier Henry:** Writing – review and editing; resources; supervision; funding acquisition; formal analysis.

## FUNDING INFORMATION

This work was funded by the National Research Council of Canada (grant PR‐023‐1) and by the Natural Sciences and Engineering Research Council of Canada (grant RGPIN/4048‐2021 and stipend allocated to Sebastian‐Juan Reyes via the NSERC‐CREATE PrEEmiuM program).

## CONFLICT OF INTEREST STATEMENT

The authors declare no conflict of interest.

## Supporting information


**Figure S1.** Daily feed volume profile of F (standard feed) and F+ (high feed) regimens. The fixed feed volume percentages (feed volume/Current medium volume) for F+ were designed to follow the increase and plateau of a cell culture run to mimic dynamic cellular kinetics. Conversely the fixed feed volume percentages (feed volume/Initial medium volume) for the F regimen were designed to be kept relatively constant across the production process. Feed additions are divided by the days between feeding events to gauge a representative feed per day plot.
**Figure S2.** Viability profile of 40% versus 60% DO at air cap of 4.2 L/H. Increased DO level demonstrates an increase in viability.
**Figure S3.** DO profiles of 40%, 60% and 90% DO conditions. Red represents the 40% DO, Green represents 60% DO and black represents 90% DO.
**Figure S4.** Amino acid profiles for two feed regimens. (A) Feed volume calculated based on current volume with bolus addition—CV [Bolus], (B) Feed volume calculated based on current volume using pump dosing—CV [Pump]. Measurement of amino acid concentrations correspond to sampling before feed additions.
**Figure S5.** Conductivity measurements of bolus and slow pump feed additions. Blue line represents pump feed addition while the maroon line represents bolus feed addition. Maroon measurements taken from control process that was temperature shifted at 0 dpi (as evidenced by the decrease in conductivity at this time point). Blue measurements taken from capacitance fed process that was temperature shifted at 2 dpi (as evidenced by the decrease in conductivity at this time point). For the bolus feed addition (maroon), each feeding event is accompanied by a step wise increase in conductivity measurements. Conversely, slow bolus additions demonstrate a constant linear increase in conductivity.
**Figure S6.** Gassing profile of a standard regimen culture fed in a bolus fashion. Green represents the DO %. Black represents the air sparging indicative of the Air Cap. Red represents on demand oxygen sparging once air cap has been reached. Air Cap commonly observed to be saturated by −1 dpi and increased oxygen requirements observed to happen in the late production phase of the culture (5–10 dpi).
**Figure S7.** Glucose consumed per day profile (GCPD) of feeding strategies study. A decrease in volumetric glucose consumption is observed to occur post induction (0 dpi). Subsequent increase glucose consumption occurs late in the production phase (4–14 dpi).
**Figure S8.** Titer profile of feeding regimens study. Rapid increase in protein concentration is observed to happen late in the production phase (7–14 dpi). Error bars represent the coefficient of variance associated to each variable.

## Data Availability

The data that support the findings of this study are available from the corresponding author upon reasonable request.
