# Supplementary material for: CHO stable pool fed‐batch process development of SARS‐CoV‐2 spike protein production: Impact of aeration conditions and feeding strategies
Source: Biotechnol Prog. 2024 Sep 27;41(1):e3507. doi: 10.1002/btpr.3507 (PMC11831418; doi:10.1002/btpr.3507)
Supplement: Supplementary file 1 — Figure S1. Daily feed volume profile of F (standard feed) and F+ (high feed) regimens. The fixed feed volume percentages (feed volume/Current medium volume) for F+ were designed to follow the increase and plateau of a cell culture run to mimic dynamic cellular kinetics. Conversely the fixed feed volume percentages (feed volume/Initial medium volume) for the F regimen were designed to be kept relatively constant across the production process. Feed additions are divided by the days between feeding events to gauge a representative feed per day plot. Figure S2. Viability profile of 40% versus 60% DO at air cap of 4.2 L/H. Increased DO level demonstrates an increase in viability. Figure S3. DO profiles of 40%, 60% and 90% DO conditions. Red represents the 40% DO, Green represents 60% DO and black represents 90% DO. Figure S4. Amino acid profiles for two feed regimens. (A) Feed volume calculated based on current volume with bolus addition—CV [Bolus], (B) Feed volume calculated based on current volume using pump dosing—CV [Pump]. Measurement of amino acid concentrations correspond to sampling before feed additions. Figure S5. Conductivity measurements of bolus and slow pump feed additions. Blue line represents pump feed addition while the maroon line represents bolus feed addition. Maroon measurements taken from control process that was temperature shifted at 0 dpi (as evidenced by the decrease in conductivity at this time point). Blue measurements taken from capacitance fed process that was temperature shifted at 2 dpi (as evidenced by the decrease in conductivity at this time point). For the bolus feed addition (maroon), each feeding event is accompanied by a step wise increase in conductivity measurements. Conversely, slow bolus additions demonstrate a constant linear increase in conductivity. Figure S6. Gassing profile of a standard regimen culture fed in a bolus fashion. Green represents the DO %. Black represents the air sparging indicative of the Air Cap. Red repres [file BTPR-41-e3507-s001.docx]

**Annex**

**
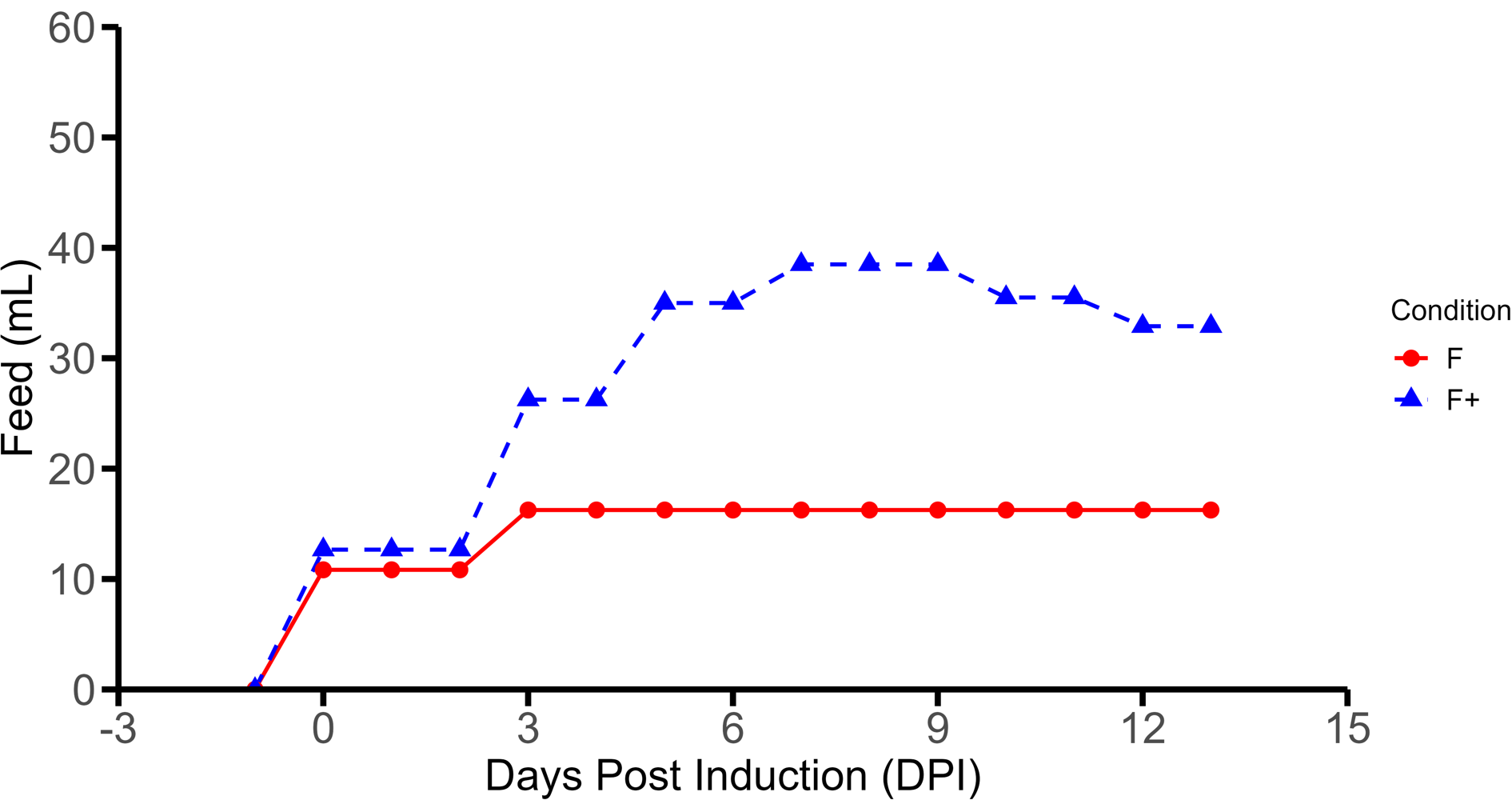
**

**FIGURE 1S** Daily feed volume profile of F (standard feed) and F+ (high feed) regimens.

The fixed feed volume percentages (feed volume/Current medium volume) for F+ were designed to follow the increase and plateau of a cell culture run to mimic dynamic cellular kinetics. Conversely the fixed feed volume percentages (feed volume/Initial medium volume) for the F regimen were designed to be kept relatively constant across the production process. Feed additions are divided by the days between feeding events to gauge a representative feed per day plot.


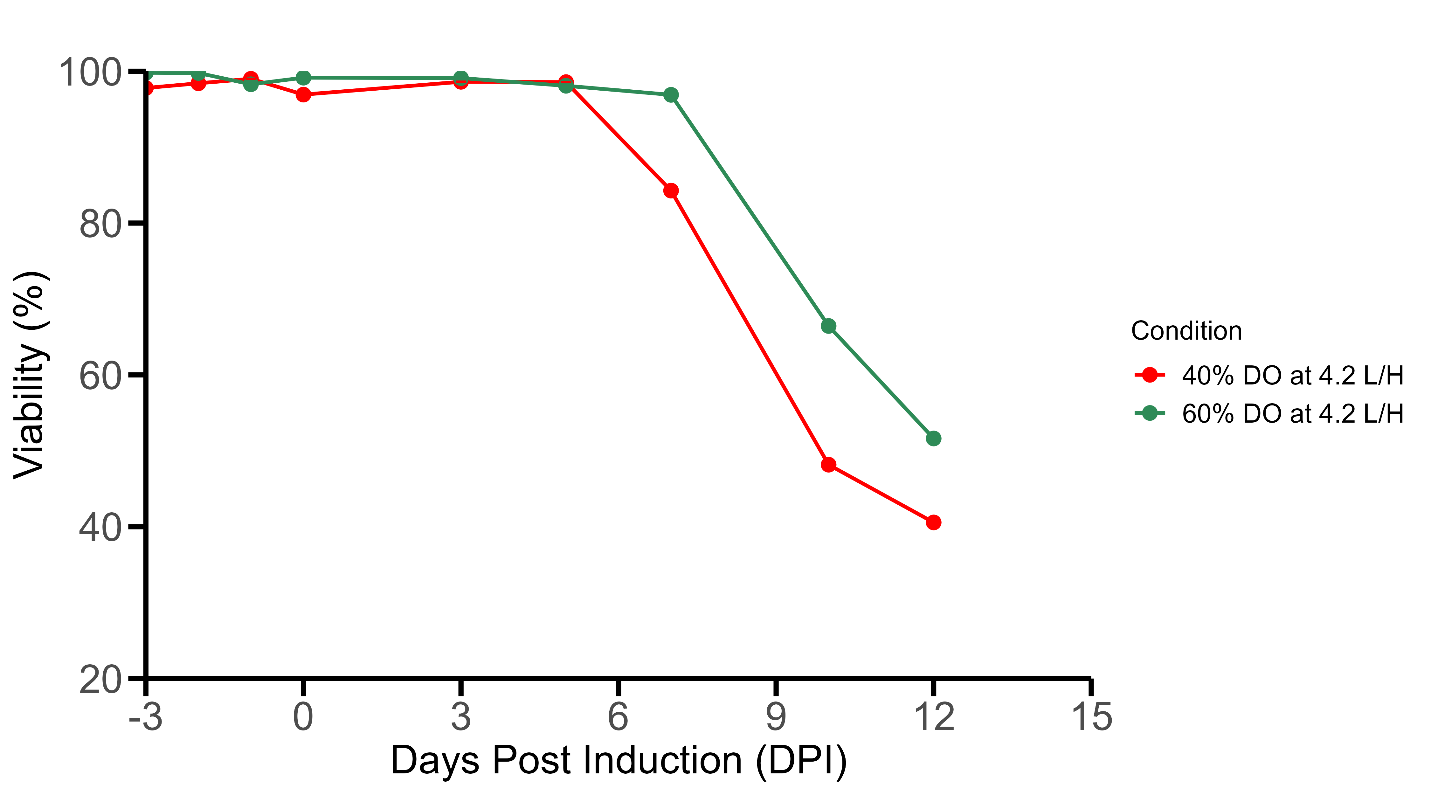


**FIGURE 2S** Viability profile of 40% vs 60% DO at air cap of 4.2 L/H. Increased DO level demonstrates an increase in viability.


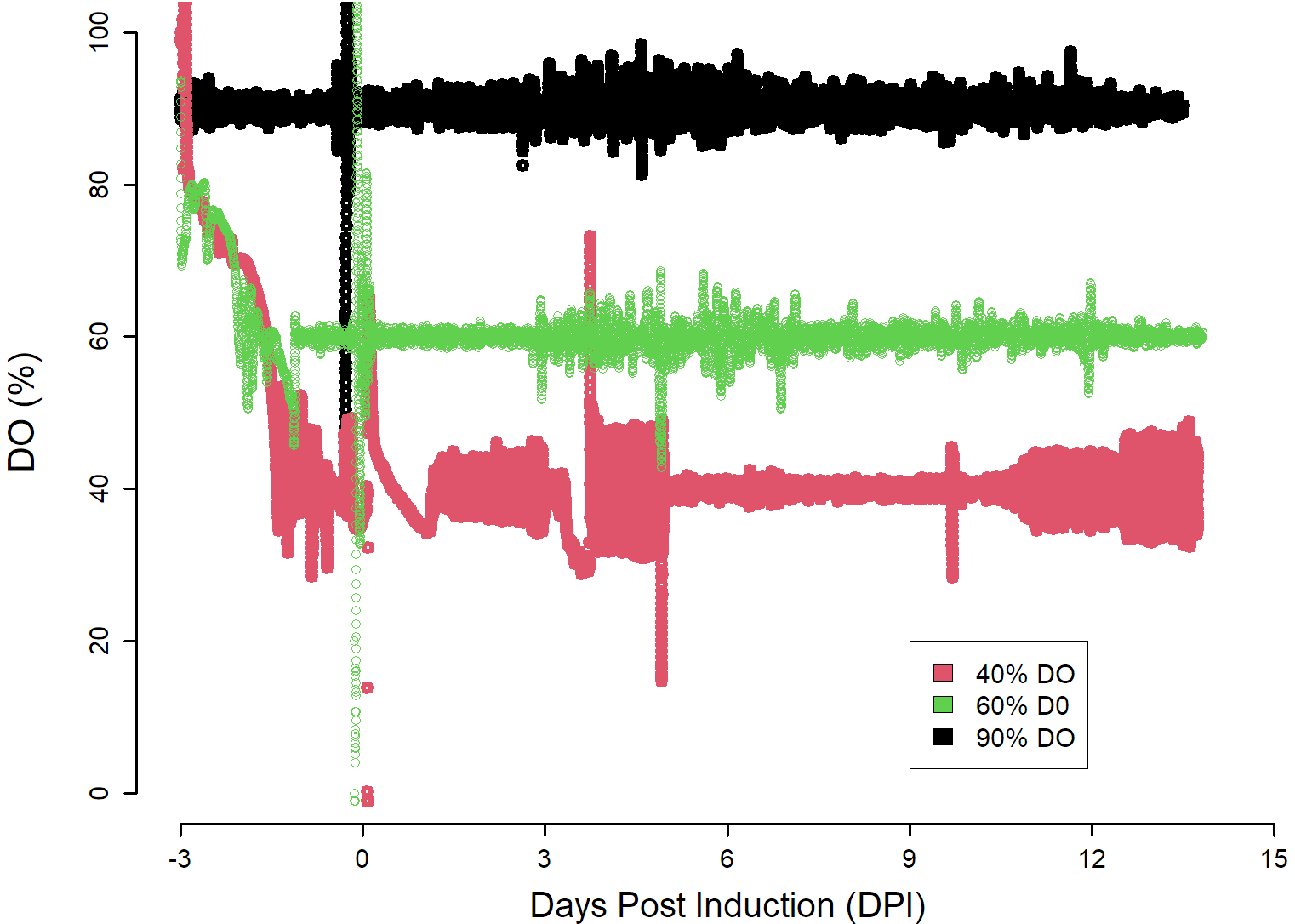


**FIGURE 3S** DO profiles of 40%, 60% and 90% DO conditions. Red represents the 40% DO, Green represents 60% DO and black represents 90% DO.

A

B

**FIGURE 4S** Amino acid profiles for two feed regimens. A) Feed volume calculated based on current volume with bolus addition - CV [Bolus], B) Feed volume calculated based on current volume using pump dosing - CV [Pump]. Measurement of amino acid concentrations correspond to sampling before feed additions.

***
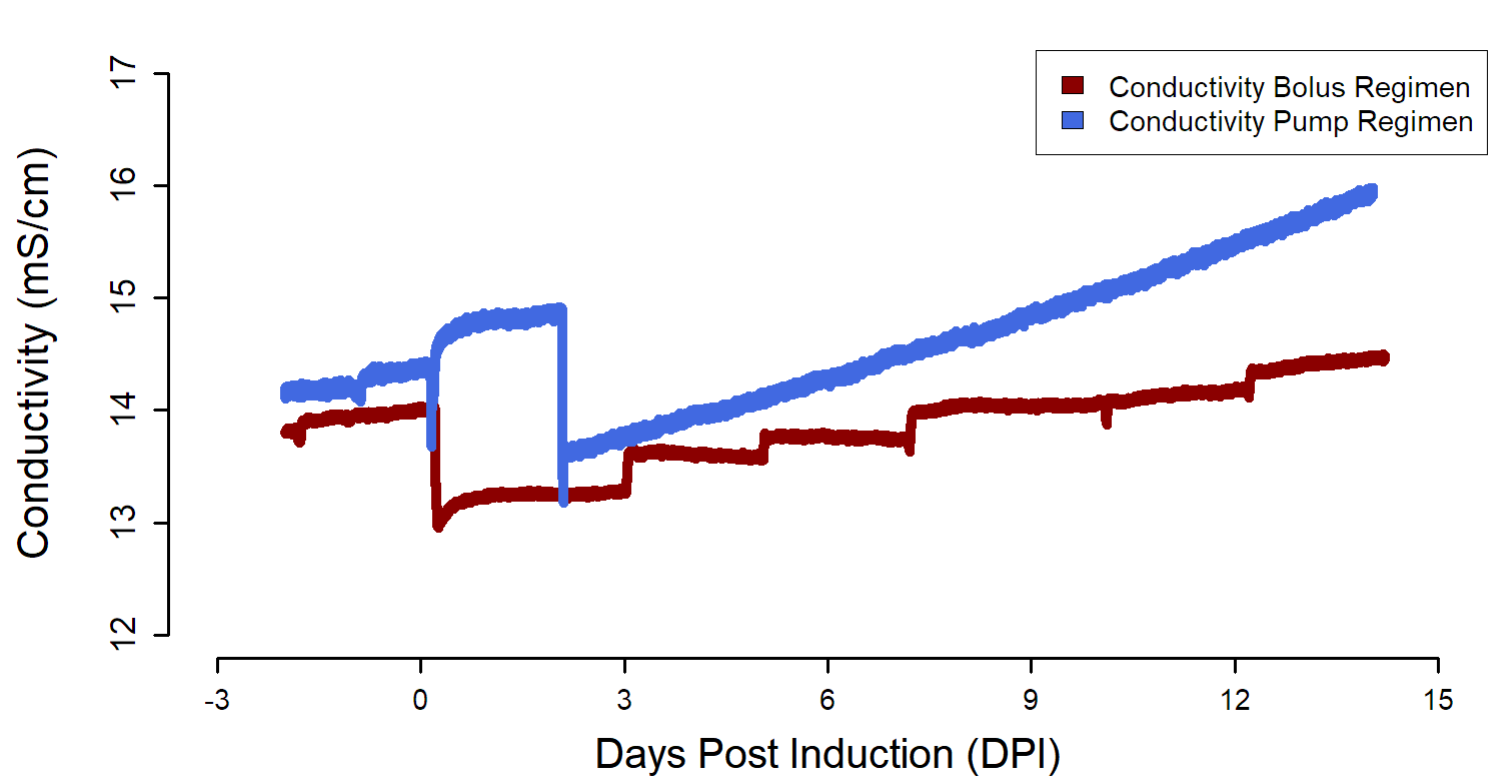
***

**FIGURE 5S** Conductivity measurements of bolus and slow pump feed additions. Blue line represents pump feed addition while the maroon line represents bolus feed addition. Maroon measurements taken from control process that was temperature shifted at 0 dpi (as evidenced by the decrease in conductivity at this time point). Blue measurements taken from capacitance fed process that was temperature shifted at 2 dpi (as evidenced by the decrease in conductivity at this time point). For the bolus feed addition (maroon), each feeding event is accompanied by a step wise increase in conductivity measurements. Conversely, slow bolus additions demonstrate a constant linear increase in conductivity.


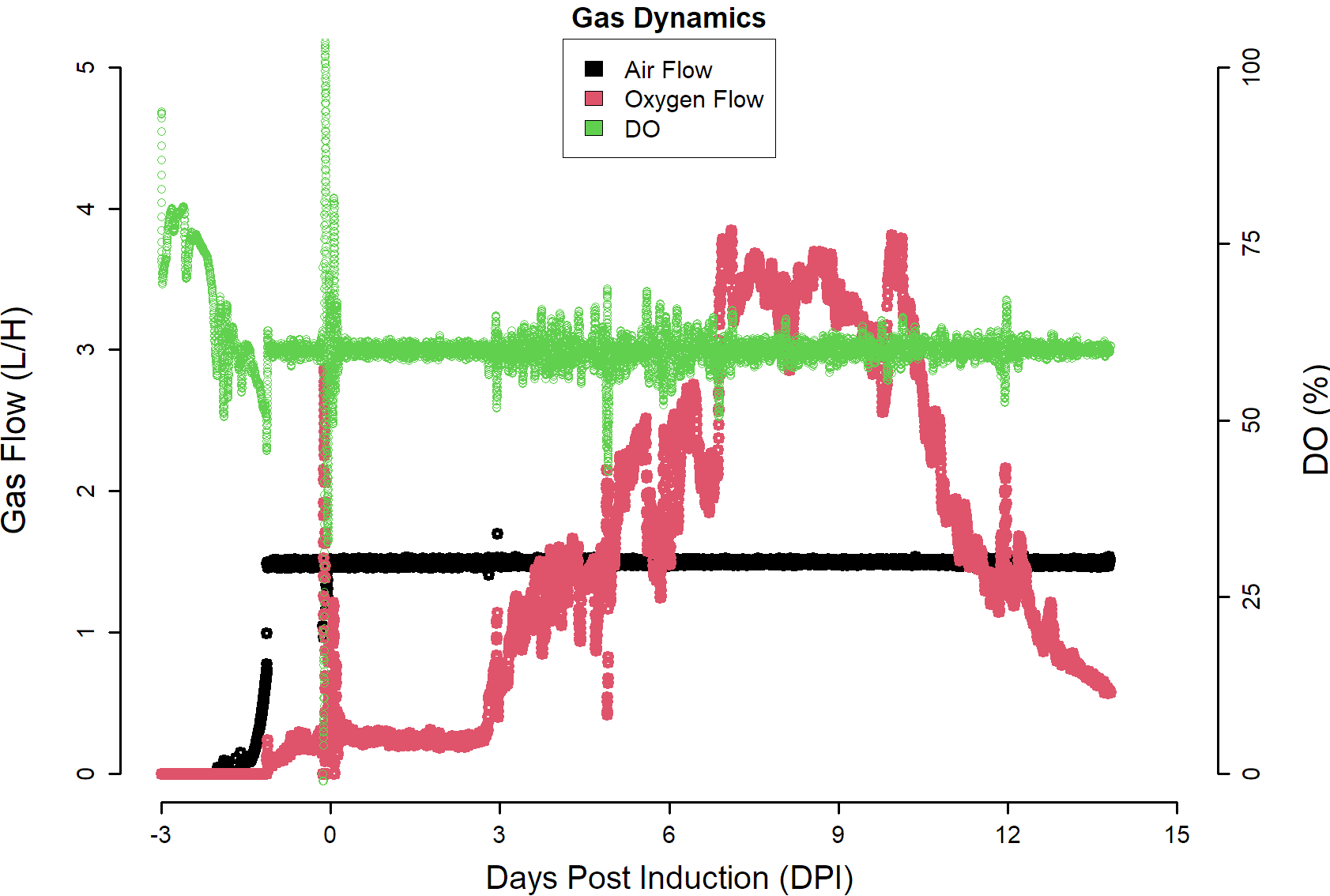


**FIGURE 6S** Gassing profile of a standard regimen culture fed in a bolus fashion. Green represents the DO %. Black represents the air sparging indicative of the Air Cap. Red represents on demand oxygen sparging once air cap has been reached. Air Cap commonly observed to be saturated by -1 dpi and increased oxygen requirements observed to happen in the late production phase of the culture (5-10 dpi).


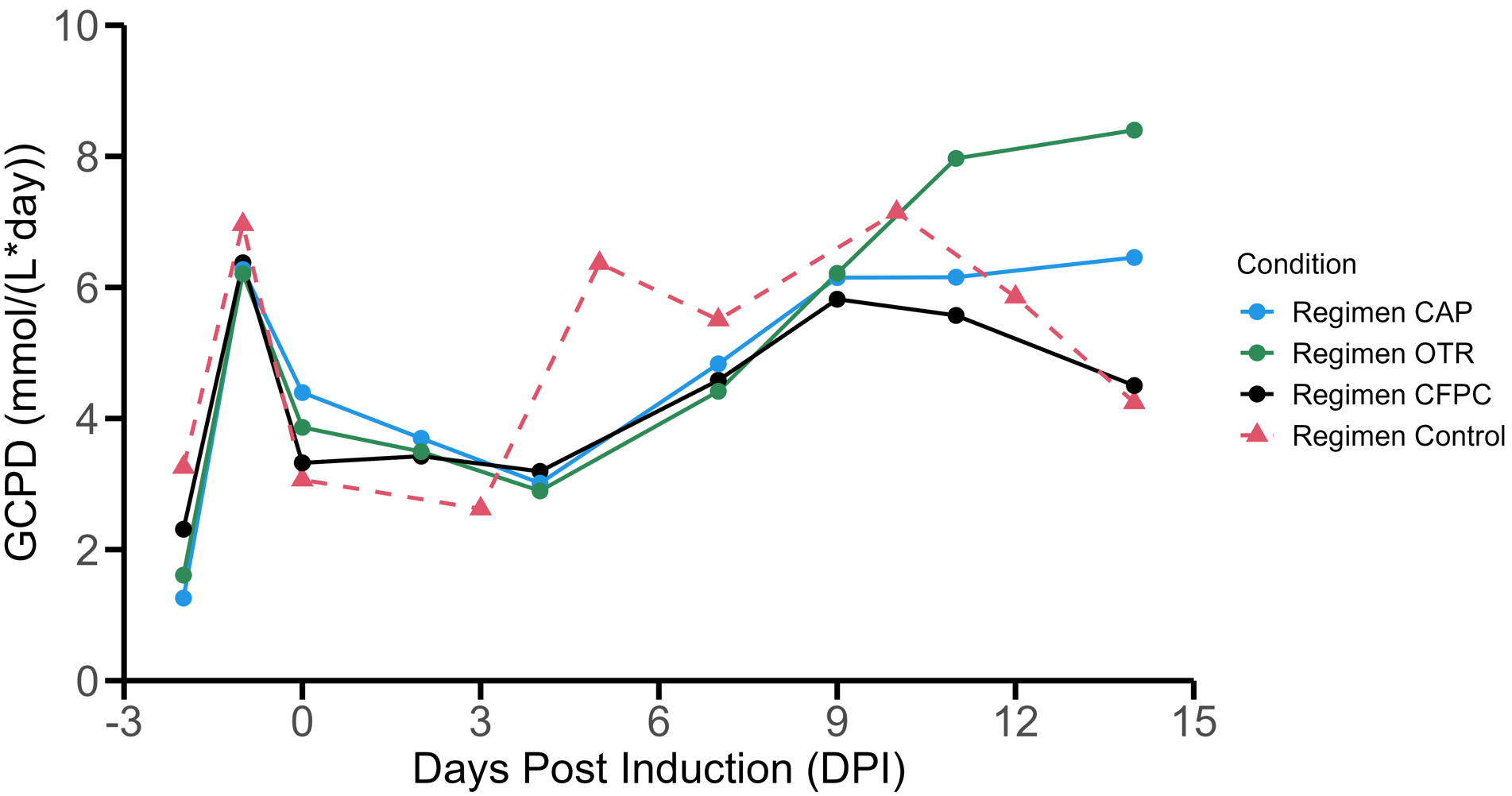


**FIGURE 7S** Glucose consumed per day profile (GCPD) of feeding strategies study. A decrease in volumetric glucose consumption is observed to occur post induction (0 dpi). Subsequent increase glucose consumption occurs late in the production phase (4-14 dpi).

**
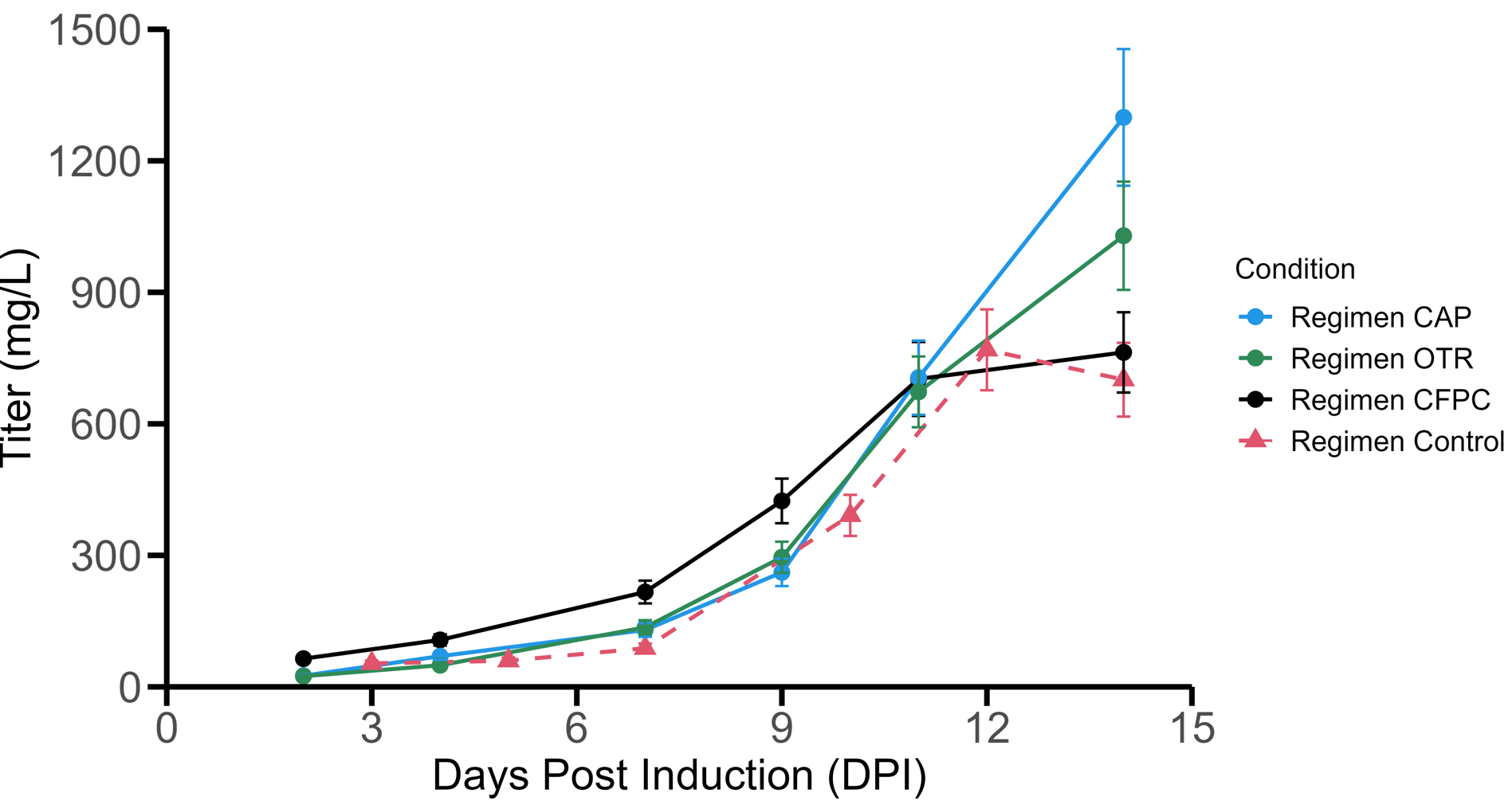
**

**FIGURE 8S** Titer profile of feeding regimens study. Rapid increase in protein concentration is observed to happen late in the production phase (7 - 14 dpi). Error bars represent the coefficient of variance associated to each variable.
